# An *In-Silico* Study on the Design of Biological Controllers for Sepsis Regulation

**DOI:** 10.1021/acsomega.5c12020

**Published:** 2026-03-11

**Authors:** Derrick Dankwa, Syeda Simra Shoaib, Leopold N. Green, Xun Tang

**Affiliations:** † Weldon School of Biomedical Engineering, 311308 Purdue University, West Lafayette, Indiana 47905, United States; ‡ Cain Department of Chemical Engineering, 5779 Louisiana State University, Baton Rouge, Louisiana 70803, United States

## Abstract

Macrophages are versatile innate immune cells that can dynamically shift between proinflammatory (M_1_) and anti-inflammatory (M_2_) states to balance immune defense and tissue repair, in response to local microenvironment cues. In sepsis, disrupted macrophage function impairs this balance, reducing pathogen clearance and increasing tissue damage. To address this, we developed a mathematical model integrating ordinary differential equations (ODEs) and a feedback control framework to design targeted interventions that promote healing. Grounded in the current knowledge of immune cell behavior and signaling, our model highlights macrophage-mediated regulation as a critical driver of infection outcomes. With model-based analysis and a biological understanding about the system dynamics, we designed IL-6-responsive feedback controllers to enhance M_1_ macrophage-driven pathogen clearance. Simulation results confirmed the efficacy of the controllers in regulating the septic conditions, showing a success of up to 95% in resolving infections when regulating multiple reactions simultaneously. Numerical analysis further demonstrated the robustness of the controllers to biological variabilities and the presence of a secondary infection. We anticipate findings from this work to guide future efforts in designing biological controllers to modulate sepsis-induced inflammation toward a regulated and pro-resolving state.

## Graphical abstract

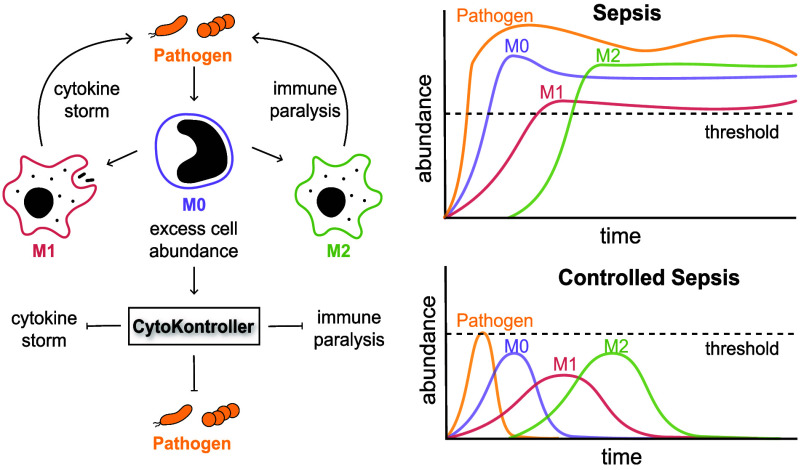

## Introduction

Sepsis is a life-threatening condition caused by a dysregulated immune response to infection that claims over 11 million lives annually, accounting for nearly 20% of global deaths.^1^ Its progression is described as a biphasic immune trajectory: an initial hyper-inflammatory phase followed by prolonged immunosuppression, driven by an imbalance of pro- and anti-inflammatory cytokines.^2^ This dynamic, which often involves elevated IL-6 levels, could lead to immune paralysis, a state where high levels of both pro-inflammatory M_1_ and anti-inflammatory M_2_ macrophages coexist, which impairs pathogen clearance and promotes tissue damage.^2,3^ As a result, it would create a nonclassical local equilibrium in the immune response, enabling virulent pathogens to evade host defenses.^4^ Therefore, to prevent organ failure and improve repair outcomes, it is critical to ensure early prediction and regulation of immune paralysis.^5^

In early sepsis, neutrophils rapidly migrate to the infection sites, triggered by pathogen-associated molecular patterns (PAMPs) binding to receptors on the resident cells.^6,7^ Neutrophils phagocytose pathogens, producing antimicrobial toxins, and undergo apoptosis postclearance, releasing cytokines such as IL-6, TNF-α, and IL-1β, to recruit monocytes (M_o_) within hours.^8,9^ As a pleiotropic cytokine, IL-6 can amplify inflammation, influence neutrophil apoptosis, and drive macrophage polarization via classical and trans-signaling pathways,^10^ thus playing a critical role in regulating the dynamics of sepsis. While controlled inflammation is essential for pathogen clearance,^11^ studies revealed that dysregulated IL-6 signaling can trigger a cytokine storm,^12,13^ leading to macrophage paralysis, where impaired M_1_-to-M_2_ switching would reduce clearance efficiency and exacerbate tissue necrosis.^14,15^

Macrophages are highly adaptable immune cells that can polarize into pro-inflammatory (M_1_) or anti-inflammatory (M_2_) phenotypes, or exist on a spectrum between these states, driving the progression and resolution of inflammation.^16,17^ In healthy responses, M_1_ macrophages would ingest apoptotic neutrophils, activating TGF-β to promote M_2_ polarization, which suppresses pro-inflammatory cytokines and aids healing.^18^ However, in sepsis, unchecked IL-6 disrupts this balance, sustaining high M_1_ and M_2_ levels, paralyzing macrophage function, and creating a microenvironment conducive to systemic inflammation.^2,19^ These findings suggest that IL-6 can be a predictive biomarker of sepsis, however, its dual role in protective and pathological responses demands precise and context-dependent regulation.^2,20^

Immunological dynamics can be probed with experimental approaches.^21,22^ However, due to the complexity of the process, such as the nonlinear signaling pathway and feedback loops, experiment-based investigations can be costly. Alternatively, mathematical modeling has emerged as a powerful complementary approach.^23,24^ Biological models for studying systems biology dynamics are broadly categorized into agent-based models (ABMs) and population-level models.^25,26^ ABMs incorporate randomness, spatial structure, and individual variability to capture more biological variances, but they would require significant computational resources and extensive data for parametrization, which could suffer from analytic tractability.^27^ In contrast, population-level models such as ordinary differential equation (ODE)-based models, normally assume homogeneous mixing and focus on population-level of dynamics, thus having the potential advantage of reduced computational demands while sacrificing individual-level variations and details.^24^ Due to their simplicity and ability to capture the essential immune dynamics, ODE-based models have been widely used in immunological modeling.^23,26,28^

In this study, we focus on how to harness system dynamics to guide the design of biological controllers for sepsis regulation. While recent advancements in systems medicine have revealed design principles for therapeutic circuits to improve immune responses in chronic conditions such as impaired wound healing and excessive scarring,^25,25,29^ here we present a model-based framework that integrates biological understanding, control theory, and synthetic biology, as a systematic approach to designing biological controllers for biosystem regulation. Specifically, we first developed a mechanistic ODE model of sepsis, focusing on macrophage polarization and IL-6-mediated signaling, inferred from literature understanding and parametrized with published data. With model-based analysis, we then designed eight feedback controllers, realizable with synthetic gene circuits, with each leveraging the IL-6 level as the feedback to regulate immune targets identified through sensitivity analysis. Simulations demonstrate that a controller that regulates M_1_ only can yield a success rate of 85% in reverting an originally septic process, and a controller that features simultaneous regulation of both M_1_ and pathogen could further improve the efficacy with a success rate of 95%. Robustness evaluation with respect to parameter uncertainty and secondary pathogen induction further suggests the reliability of the proposed controllers, offering a promising strategy to mitigate immune paralysis and bacterial invasion. We anticipate findings from this work to guide the future experimental development of adaptive, feedback-based therapies for sepsis.

## Results and Discussion

### Sepsis Model Development

The ODE model in this study was developed based on the Law of Mass Action described in eqs 1–9 to simulate the sepsis process outlined in Figure 1A, by capturing interactions among key innate immune cells (monocytes, M_1_ and M_2_ macrophages, and neutrophils) and representative cytokines (IL-6 and TGF-β). While numerous cytokines influence sepsis, we focused on IL-6 and TGF-β due to their well-characterized roles in upstream gene regulation and downstream immune modulation across innate immune response phases.

**Figure 1:**
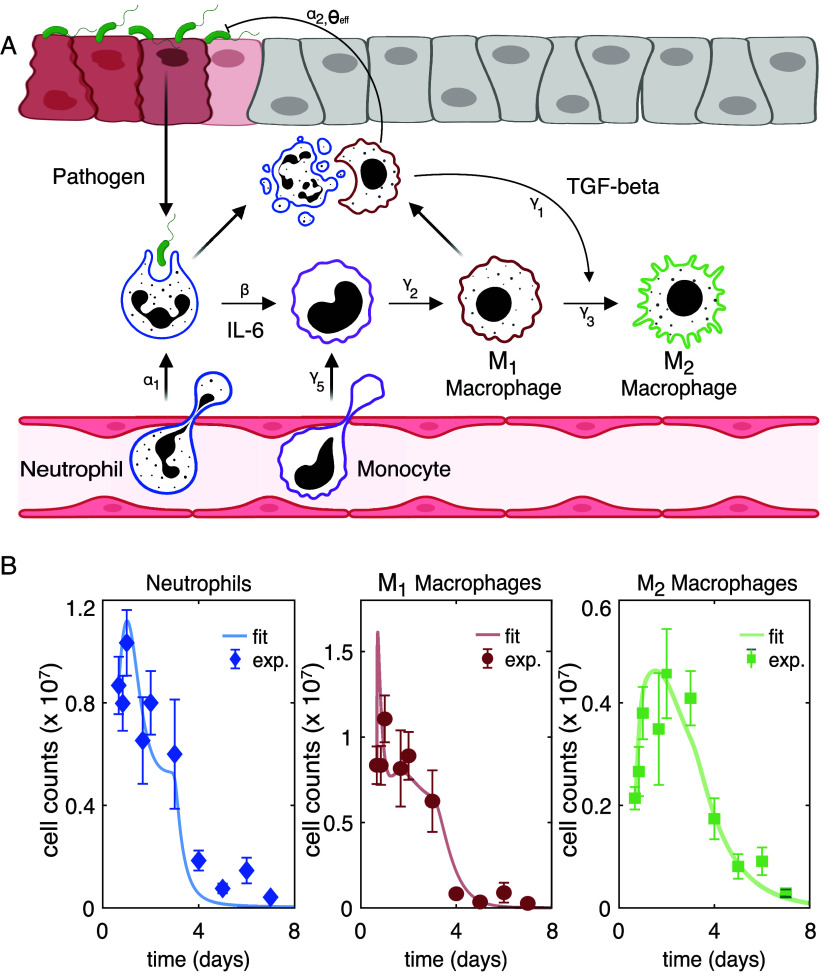
(A) Overview of innate immune cell dynamics following pathogen invasion. (B) ODE model is parametrized with experimental measurements to extract biologically relevant kinetic parameters by fitting to the reported neutrophil, M_1_, and M_2_ cell count dynamics. Solid lines are model predictions, and dotted plots are for experimental measurements. Fitted parameter values are reported in Table S1.

Prior to infection, the system is assumed to exist in a low-pathogen state with minimal immune cell activation. Pathogen invasion initiates a cascade of innate immune responses that progress on distinct but coupled time scales (eq 1). Generally, neutrophils are the first immune cells recruited in response to microbial byproducts, such as pathogen-associated molecular patterns (PAMPs), by recognizing pathogen recognition receptors on resident epithelial and circulating immune cells. This recruitment is primarily driven by pathogen sensing, which is modeled as a saturating Hill-type function parametrized by the maximal recruitment rate α_1_, half-saturation constant *K*_*n*_, and Hill coefficient *n* as in eq 2. This Hill-type function framework captures the rapid neutrophil influx at low-to-moderate pathogen levels and saturation at higher burdens due to receptor and signaling constraints. Upon activation, neutrophils’ primary role shifts to phagocytosis, actively engulfing and digesting pathogens by generating toxic chemicals that have antimicrobial activity inside phagosomes. We modeled these dynamics as a mass-action interaction proportional to pathogen abundance and neutrophil density scaled by the clearance rate γ_4_ in eq 2. The neutrophil–pathogen interaction is included only in the pathogen equation with a clearance term γ_4_, without a corresponding term in the neutrophil equation, due to the assumption that pathogen killing by neutrophils does not cause neutrophil depletion on the acute inflammatory time scale. Instead, neutrophil depletion is governed independently by a basal decay rate μ_1_ and cytokine-mediated turnover, particularly IL-6–dependent mechanisms that couple recruitment and activation to signal transducer and activator of transcription 3 (STAT3)-mediated survival signaling, as well as cytokine-induced exhaustion and consumption scaled by parameter γ_5_. This design maintains a separation between the antimicrobial function of neutrophils and their population-level dynamics, where neutrophil recruitment is constrained by a global carrying capacity *C*_max_ (eq 8) dependent on the total myeloid burden, to prevent unbounded expansion and to incorporate spatial limitations, bone marrow constraints, and immune exhaustion, following Torres et al.^23^ (eq 2).

Monocyte recruitment occurs subsequently via IL-6–mediated signaling at a rate γ_5_, linking early neutrophil-driven inflammation to later macrophage expansion. Monocytes differentiate into pro-inflammatory M_1_ macrophages at rate γ_2_ and decays at rate μ_2_ (eq 3). M_1_ macrophages undergo intrinsic decay at a rate of μ_3_ and are negatively regulated by TGF-β through a suppression term scaled by γ_3_, capturing the cytokine-driven phenotypic deactivation and functional inhibition (eq 4). Pathogen clearance by M_1_ macrophages is modeled as a bilinear interaction with rate α_2_, modulated by a nonlinear effector function, effector (eq 9), with activation threshold θ_eff_ for pathogen regulation by M_1_ and steepness parameter κ (eq 1). This effector formulation ensures minimal antimicrobial activity below the threshold and rapidly increasing efficacy once activated, reflecting the observed switch-like behavior of macrophages. TGF-β drives the polarization of M_1_ to anti-inflammatory M_2_ macrophages at a rate of γ_3_, promoting a regulatory, tissue-repair phenotype. M_2_ macrophages decay at a rate μ_4_, representing terminal differentiation and resolution-associated clearance (eq 5).

IL-6 production is driven by neutrophil availability, scaled by production rate β, and decays at a rate δ_1_, reflecting its rapid induction and relatively short half-life. TGF-β production is driven by sustained interactions between M_1_ macrophages and neutrophils, scaled by γ_1_, and decays at a slower clearance rate δ_2_, thereby establishing a delayed negative feedback loop that suppresses M_1_ activity and favors M_2_ accumulation, simulating its role as a delayed regulatory signal, primarily functioning as an immunosuppressant in inflammation resolution and tissue repair (eq 7). The detailed description of each parameter is provided in the Supporting Information Table S1.

1 $$\frac{d\left[p\mathrm{athogen}\right]}{\mathrm{dt}}=\alpha_{2}\left[M_{1}\right]\left[p\mathrm{athogen}\right]\cdot e\mathrm{ffector}\left(M_{1},\theta_{eff}\right)-\gamma_{4}\left[n\mathrm{eutrophil}\right]\left[p\mathrm{athogen}\right]-\delta_{3}\left[p\mathrm{athogen}\right]$$

2 $$\frac{d\left[\mathrm{neutrophil}\right]}{\mathrm{dt}}=\alpha_{1}\frac{{\left[\mathrm{pathogen}\right]}^{n}}{{\left[\mathrm{pathogen}\right]}^{n}+K^{n}}\cdot c\mathrm{apacity}-\gamma_{5}\left[\mathrm{neutrophil}\right]\left[IL-6\right]-\mu_{1}\left[\mathrm{neutrophil}\right]$$

3 $$\frac{d\left[m\mathrm{onocyte}\right]}{\mathrm{dt}}=\gamma_{5}\left[n\mathrm{eutrophil}\right]\left[IL-6\right]-\gamma_{2}\left[m\mathrm{onocyt}e\right]-\mu_{2}\left[m\mathrm{onocyte}\right]$$

4 $$\frac{d\left[M_{1}\right]}{\mathrm{dt}}=\gamma_{2}\left[m\mathrm{onocyte}\right]-\gamma_{3}\left[M_{1}\right]\left[TGF-\beta \right]-\mu_{3}\left[M_{1}\right]$$

5 $$\frac{d\left[M_{2}\right]}{\mathrm{dt}}=\gamma_{3}\left[M_{1}\right]\left[TGF-\beta \right]-\mu_{4}\left[M_{2}\right]$$

6 $$\frac{d\left[IL-6\right]}{\mathrm{dt}}=\beta \left[n\mathrm{eutrophil}\right]-\delta_{1}\left[IL-6\right]$$

7 $$\frac{d\left[TGF-\beta \right]}{\mathrm{dt}}=\gamma_{1}\left[M_{1}\right]\left[n\mathrm{eutrophil}\right]-\delta_{2}\left[TGF-\beta \right]$$

**Capacity function**

8 $$c\mathrm{apacity}=1-\frac{\left[n\mathrm{eutrophil}\right]+\left[m\mathrm{onocyte}\right]+\left[M_{1}\right]+\left[M_{2}\right]}{C_{\max}}$$

**Effector function**

9 $$e\mathrm{ffector}\left(M_{1},\theta_{eff},k\right)=\frac{2}{1+e^{-k\left(M_{1}-\theta_{eff}\right)}}-1$$

To ensure biological relevance, the model was parametrized by fitting to *in vivo* time-course data obtained from a murine thioglycollate-induced peritonitis model, a well-established platform for studying acute inflammatory responses and immune cell trafficking, reported in Torres et al.,^23^ using MATLAB *fmincon* to simultaneously fit the neutrophil, M_1_, and M_2_ dynamics. Figure 1B shows the comparison of the predicted (solid lines) and the reported experimental measurements (dotted plots) of the neutrophil, M_1_, and M_2_ dynamics, confirming the model’s accuracy. The fitted parameter values are provided in Table S1 and are referred to as nominal values for subsequent analyses. A summary on the experiments performed in Torres et al.^23^ and the identification of the initial guesses for the model parameters are also provided in SI document.

### Global Sensitivity Analysis Reveals Divergent Immune Dynamics

To understand the system dynamics under different immune responses and to explore conditions that could potentially lead to immune paralysis, we performed a global sensitivity analysis by varying all 19 model parameters simultaneously over [0.1–10] times their Nominal values. For statistical significance, a total of 1000 parameter sets were randomly sampled with a uniform distribution for the analysis. Specifically, each simulation was initiated with a pathogen concentration of 20 a.u., serving as a proxy to early stage pathogenesis. A simulation with a pathogen concentration of 2 or less by Day 8 is then classified as “Acute healing”, as it represents a 90% reduction from the initial pathogen concentration and indicates a significant pathogen clearance, a criterion inferred from Karthika et al.^30^ Otherwise, the simulation is denoted as “septic healing”, the condition that we aim to regulate.

Figure 2 summarizes the results of the 1000 simulations, where 90.6% presented acute healing dynamics and 9.4% demonstrated persistent pathogen or sepsis. This finding aligns with clinical trends where most infections would clear up, but a significant minority, especially in high-risk patients, would advance to sepsis.^1^ The solid green traces and shaded area represent the acute healing scenarios post microbial challenge, whereas the solid red traces represent the average of all septic healing simulations, with the shaded area representing the span of the entire septic dynamics. Several distinct dynamics were observed between the acute and septic healing processes. The M_o_, M_1_, M_2_, Pathogen, and IL-6 septic healing dynamics all showed a significantly higher maximum level compared to that of the acute healing conditions over time, demonstrating the prolonged healing dynamics. The average M_o_ cell count in both septic and acute healing conditions showed a drastic reduction after the invasion of the pathogen, with the septic M_o_ cell count ending at a relatively higher level. The average acute healing M_1_ cell count dynamics showed a consistent decreasing trend over time, with a consistently low level of M_2_ cell count and a sustained pathogen level succeeding the initial increase after pathogen invasion. In contrast, the average M_1_ cell count in the septic healing conditions showed an overall increase trend, accompanied by a similar trend in both M_2_ and pathogen. Comparison of IL-6 dynamics also demonstrated a significantly higher level in the septic healing condition, aligning with previous findings that unchecked high IL-6 level could be a biomarker for the onset of sepsis.^2,19^

**Figure 2:**
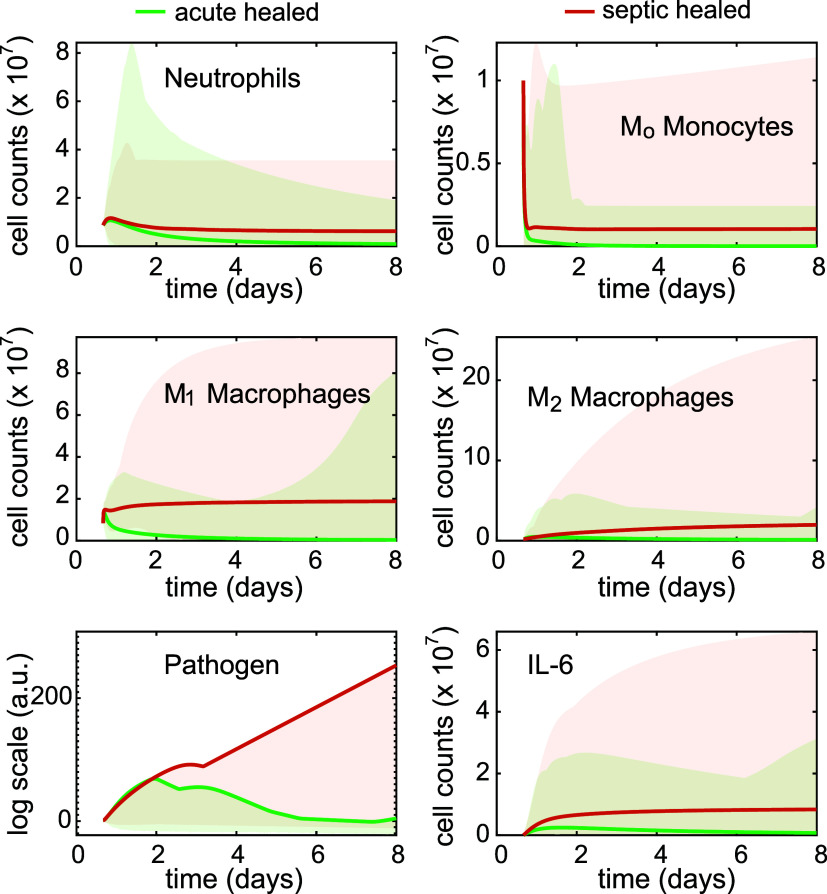
Global sensitivity analysis reveals distinct dynamics between acute (green) and septic (red) healing processes, especially in M_1_, M_2_, and Pathogen, suggesting potential regulation targets for the controller design. The bold line represents the average of the 1000 simulations, and the shaded regions indicate the minimum to maximum values across the simulations.

These “septic” parameter regimes signify critical failures in immune regulation, and the dramatically different dynamics of M_o_, M_1_, M_2_, and pathogen further suggest that these species could be potential regulatory points for intervention. Based on these findings, we then sought to design our feedback controllers to regulate the septic healing dynamics to acute healing.

### Feedback Controller Design

A typical feedback control scheme includes: sensing, which monitors the process dynamics as feedback to facilitate control decision-making; a control policy, which dictates how to regulate the dynamics; and actuation, which executes the control action to influence the process dynamics. In our system, the real-time IL-6 level is monitored as feedback to inform the regulation of the healing process, and this can be achieved with synthetic gene circuits by implementing tunable thresholds via sequestration. For example, the IL-6 neutralization of Siltuximab reported in^25^. The difference between measured and target (i.e., threshold) IL-6 levels is then scaled by the gene circuit (i.e., control policy, tunable by designing the circuit components) to affect the target immune components dynamics (i.e., actuation), mimicking the proportional control, where the control action is proportional to the difference between the output and the target. Based on this, we then proposed four single-species regulation controllers, named as CytoKontrollers, that monitor the IL-6 level and regulate the activity of a single immune component, with each featuring a Hill-type activation function to represent the dynamics of the gene circuit. Note that, in all controllers, α is the controller strength, similar to the controller gain in control theory, *n* is the cooperativity of the Hill function, and *K*_d_ is the controller activation threshold, and all these three controller parameters are tunable by designing the realization of the gene circuit, such as promoter strength.

The first controller, CytoKontroller 1, monitors IL-6 level and regulates the M_1_ macrophage population, by manipulating the degradation rate of M_1_, according to ${\mu_{3}}^{cont}=\mu_{3}\left(1+\alpha \frac{{IL-6}^{n}}{{K_{d}}^{n}+{IL-6}^{n}}\right)$ . This design is based on the observation from Figure 2 that M_1_ remains elevated (∼1.5–2.0 × 10^7^) in the septic conditions but drops to near zero in the acute healing conditions. In this controller, μ_3_ is the original M_1_ removal rate, and μ_3_^cont^ is the required M_1_ removal rate calculated from the controller. The controller increases M_1_ macrophage clearance when the IL-6 level exceeds the target threshold by accelerating the removal of M_1_ during severe inflammation while preserving normal dynamics in mild cases. This control mechanism can be achieved using synthetic gene circuits in macrophages or commensals that sense and respond to IL-6 levels via receptor-based sensors, triggering downstream effectors to control apoptosis or deactivation. For instance, IL-6-induced STAT3 activation could drive synthetic nucleic acid regulatory systems to express pro-apoptotic genes (e.g., Bax or caspase activators).^31^ Alternatively, RNA interference or CRISPR-based regulation could dynamically modulate transcription factors such as NF-κB in response to IL-6, fine-tuning the M_1_ macrophage lifespan to reduce excessive inflammation while maintaining host defense.

The second controller, CytoKontroller 2, was designed to act directly on the pathogen level by enhancing innate clearance capacity, according to ${\delta_{3}}^{\mathrm{cont}}=\delta_{3}\left(1+\alpha \frac{{\mathrm{IL}-6}^{n}}{{K_{d}}^{n}+{\mathrm{IL}-6}^{n}}\right)$ , where δ_3_ and δ_3_^cont^ are the original and required pathogen removal rates. This design is based on the observation that pathogens are cleared within days under acute healing conditions, whereas in septic cases, pathogens exhibit an exponential growth phase as in Figure 2. CytoKontroller 2 adjusts the pathogen clearance rate via IL-6 inflammatory levels, enabling an inflammation-driven antimicrobial response. This controller can be implemented by engineering immune cells, such as neutrophils or macrophages, with IL-6-responsive synthetic gene circuits. For example, macrophages can be designed to upregulate antimicrobial peptides (e.g., defensins or cathelicidins) when IL-6 exceeds a clinically defined threshold, enhancing pathogen clearance.^32^

The third controller, CytoKontroller 3, targets monocytes (M_o_), which are the precursors of M_1_ macrophages, according to ${\mu_{2}}^{\mathrm{cont}}=\mu_{2}\left(1+\alpha \frac{{\mathrm{IL}-6}^{n}}{{K_{d}}^{n}+{\mathrm{IL}-6}^{n}}\right)$ , where μ_2_ and μ_2_^cont^ are the original and required M_o_ removal rates, respectively. This design is based on the observation from Figure 2 that M_o_ level remains noticeably lower in the acute healing conditions as compared to that in the septic healing conditions, and that reducing M_o_ availability during intense inflammation could enhance healing by limiting M_1_ cell production. This control strategy can be implemented using a synthetic IL-6-responsive gene circuit that modulates M_o_ levels. For example, IL-6 can be sensed via the IL-6R/gp130 pathway, which activates a STAT3-inducible promoter (pSTAT3) to drive the expression of monocyte-depleting effectors. Two such actuation strategies have been proposed: (1) a suicide gene system using inducible caspase-9 (iCasp9) under pSTAT3 to trigger M_o_ apoptosis upon small-molecule dimerized administration;^33–35^ and (2) a secreted monoclonal antibody targeting CD14 to mark M_o_ cells for depletion via antibody-dependent cellular cytotoxicity (ADCC).^36,37^

The fourth controller, CytoKontroller 4, was designed to promote pro-resolving M_2_ macrophages, according to ${\gamma_{3}}^{\mathrm{cont}}=\gamma_{3}\left(1+\alpha \frac{{\mathrm{IL}-6}^{n}}{{K_{d}}^{n}+{\mathrm{IL}-6}^{n}}\right)$ , where γ_3_ and γ_3_^cont^ are the original and required M_1_-to-M_2_ differentiation rates. This controller can be implemented using a synthetic gene circuit that senses elevated IL-6 via the IL-6R/gp130 pathway, designed such that excess IL-6 would trigger the expression of IL-10 from a downstream promoter, which further drives M_2_ polarization.^38^ Alternatively, the circuit can express PPARγ (Peroxisome proliferator-activated receptor γ), a protein that upregulates M_2_ markers (e.g., IL-10 and TGF-β) while suppressing M_1_ genes (e.g., TNF-α and IL-1β),^39^ thus accelerating immune resolution and tissue repair.

### Controller Hyperparameter Optimization

Before evaluating the performance of the controllers, we first sought to optimize the controller design, by tuning the controller hyperparameters, i.e., control strength (α), cooperativity (*n*), and activation threshold (*K*_d_). Specifically, we confined α values between 0.1 and 10, *n* values between 1 and 4, and the *K*_d_ values between 0.2 and 1.5, considering the biological feasibility based on the literature. We tested a total of 168 controller parameter combinations, corresponding to α = [0.1, 0.5, 1, 2, 5, 8, 10], *n* = [1, 2, 3, 4], and *K*_d_ = [0.2, 0.4, 0.6, 0.8, 1, 1.5], on the 94 septic simulations generated from the global sensitivity analysis in Figure 2. A successful regulation is defined as the controller can revert the originally septic simulation to achieve ≥90% pathogen clearance by Day 8, and the success rate is defined as the ratio of the number of successful regulations over the total number of the originally septic simulations (i.e., 94). We then selected the parameter combination that yielded the highest success rate as the controller hyperparameters for further evaluation. Figure 3 illustrates this optimization analysis, using CytoKontroller 1 as an example.

**Figure 3:**
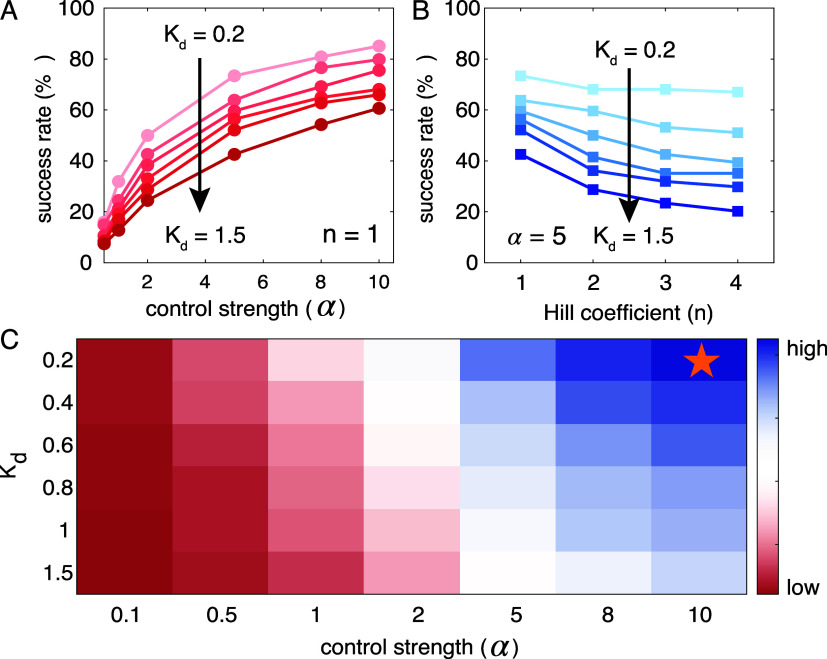
Optimization of CytoKontroller 1 by varying control strength, Hill coefficient, and activation threshold. (A) Success rate as a function of control strength and activation threshold at a fixed Hill coefficient (*n* = 1). (B) Success rate as a function of the Hill coefficient and activation threshold at a fixed control strength (α = 5). (C) Heatmap of success rates across all the tested α–*K*_d_ parameter combinations for *n* = 1, with the highest success rate achieved with α = 10 and *K*_d_ = 0.2, indicated by the yellow star. The color scheme from dark red to dark blue correlates to success rate from low to high.

Results in Figure 3A suggest that with a fixed Hill coefficient *n* value, the controller performance would increase as the control strength α increases but would decrease as the threshold *K*_d_ value increases. This further suggests that an aggressive regulation (with a higher control strength) with an early detection (with a small *K*_d_ value) of the onset of sepsis would lead to a higher chance of achieving a successful intervention. Results in Figure 3B suggest that for a fixed control strength, increasing either the Hill coefficient *n* value or the threshold *K*_d_ value would deteriorate the controller performance, as it would reduce the controller sensitivity in detecting an early onset of sepsis. Figure 3C summarizes the success rate of all tested control strengths and threshold *K*_d_ values for *n* = 1, demonstrating that a high control strength with a low threshold value would contribute to a more effective controller. Moving forward, we focus our analysis on controllers with *n* = 1, α = 10, and *K*_d_ = 0.2, as this combination yielded the highest success rate for CytoKontroller 1, as indicated by the yellow star symbol in Figure 3C.

### Controller Performance Evaluation

The performance of each CytoKontroller is then evaluated by applying the controller to regulate the 94 septic simulations identified from the global sensitivity in Figure 2, and the results are summarized in Figure 4. Black plots represent the averaged uncontrolled septic simulations; red, cyan, purple, and green plots are for the averaged controlled results with CytoKontroller 1, 2, 3, and 4, respectively.

**Figure 4:**
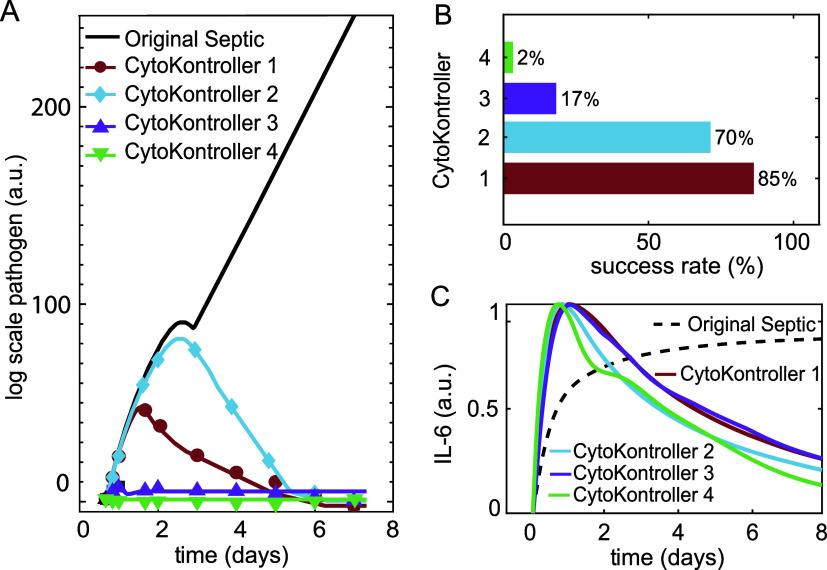
Single CytoKontroller performance evaluation. (A) Comparison of single log-scale pathogen dynamics from simulations with and without control. (B) Success rate in reverting the septic condition to acute healing with each CytoKontroller. (C) Comparison of single IL-6 dynamics from simulations with and without control. Black plots are for original septic simulation obtained from the global sensitivity analysis, red, cyan, purple, and green are for CytoKontroller 1, 2, 3, and 4, respectively.

Comparison in terms of regulation success rate in Figure 4B demonstrates that direct removal of M_1_ (CytoKontroller 1) or pathogen (CytoKontroller 2) yielded the highest efficacy, achieving success rates of 85 and 70%, respectively. Regulating the M_o_ removal rate (CytoKontroller 3) had a minor effect with a 17% success rate, while modulating the M_2_ production rate (CytoKontroller 4) showed negligible efficacy with a 2% success rate. Figure 4A,C present a comparison of a single simulation from each of the conditions. The log-scale pathogen dynamics in Figure 4A reveal that, with the effective controllers, pathogen levels increased at the beginning and then dropped dramatically in the system, aligning with the IL-6 dynamics in Figure 4C. However, with CytoKontroller 3 and 4, despite that they led to similar IL-6 dynamics and significantly reduced pathogen levels as compared to the unregulated septic conditions, they have failed to revert most of the septic healing processes. These findings reconcile clinical expectations that interventions directly targeting pathogen dynamics through modulation of its removal rate δ_3_ ^40^ or generation via M_1_ concentration μ_3_ had the most significant impact.^39^

To evaluate the robustness of the controller under immune heterogeneity in sepsis, we then focused on the best-performing single-species-based controller, CytoKontroller 1. Specifically, instead of varying all of the system parameters, we focused on the six most impactful parameters, μ_3_, μ_4_, δ_1_, γ_3_, β, and δ_3_, identified from local sensitivity analysis as summarized in the Supporting Information. For each of the 94 septic conditions from the global sensitivity analysis in Figure 2, we conducted Latin Hypercube Sampling to generate a total of 100 combinations of system parameters by introducing variations up to ±50% of the nominal value to each of the six parameters. We then calculated the success rate for each of the 94 sets of 100 simulations as an assessment to the controller robustness. For example, the success rate for septic condition 1 quantifies the ratio of the 100 simulations sampled from the Latin hypercube Sampling that have reached a more than 90% of pathogen clearance by Day 8. The histogram distribution of the 94 success rates in Figure 5A demonstrates that the controller can maintain an 82.2% success rate overall, slightly lower than the original success rate (85%), despite an up to 50% deviation in the key parameter values, confirming its robustness.

**Figure 5:**
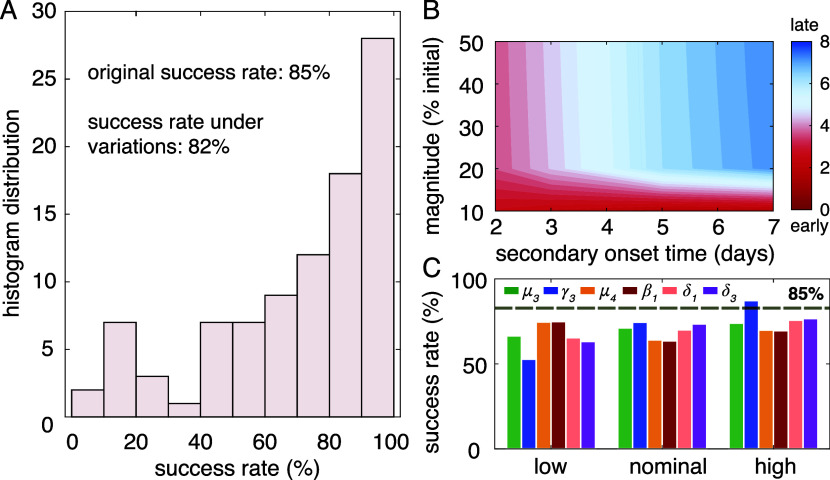
Robustness analysis of CytoKontroller 1. (A) Histogram distribution of the success rate in controlling the 94 septic simulations from the global sensitivity analysis, subjecting to ±50% of variations in the six most impactful system parameters, demonstrating an average of 82% success rate. (B) Heatmap summary of the average time needed for the controller to handle a secondary pathogen invasion after the initial pathogen load. Dark red indicates rapid elimination, and dark blue indicates prolonged healing. (C) Analyzing the success rates in terms of system parameter values, low (0.5–0.8 × Nominal), nominal (0.8–1.2 × Nominal), and high (1.2–2.0 × Nominal), suggests potential improvement with regulating multiple activities.

To assess the resilience of CytoKontroller 1 against secondary infections, we randomly selected 20 healed simulations from the CytoKontroller 1-regulated cases and introduced additional microbial challenges at levels of 10, 20, 30, 40, and 50% of the initial pathogen load, between Day 2 and Day 7 postinitial pathogen challenge to each simulation (i.e., a total of 5 × 6 = 30 combinations of secondary infection to each of the 20 healed simulations), to simulate conditions such as microbial resistance or the immunocompromised states. Out of the 30 × 20 = 600 simulations, 118 simulations ended up with a 90% initial pathogen clearance by Day 8 with the controller, yielding a 20% of success rate overall. Figure 5B presents the average time required for pathogen clearance for the 118 successful simulations as a function of induction time and load percentage magnitude. Dark red to dark blue indicates the increase of time required for infection removal. We notice several observations from these results. First, lower load of secondary pathogen infection (i.e., magnitude of ≤20%) can be rapidly eliminated regardless of the time of its introduction. Second, secondary infection introduced at earlier stages can be more rapidly eliminated across different magnitudes. These findings further highlighted the importance of early detection and intervention of the onset of sepsis for an effective intervention.

While analyzing the robustness testing results, we further noticed that elevated γ_3_ (differentiation rate of M_1_-to-M_2_) can improve the performance of the controller as indicated by Figure 5C, which presents the success rate in Figure 5A in terms of low (0.5–0.8 × Nominal), nominal (0.8–1.2 × Nominal), and high (1.2–2.0 × Nominal) values of the six tested system parameters, to understand the correlation between the parameter values and the controller efficacy. Notice that, with a high γ_3_ value (blue bar in the high category), the controller success rate surpassed the original 85% success rate of CytoKontroller 1. This observation reconciles recent studies of isoflavanoid treatments to LPS-induced sepsis,^41,42^ and further suggests that simultaneous regulation of multiple interactions could potentially further enhance the controller efficacy.

To test this hypothesis, we then designed and evaluated the performance of three Dual CytoKontroller that simultaneously regulate two reactions and one Triple CytoKontroller that regulates three reactions at the same time on the same set of septic simulations from the global sensitivity analysis.

The first dual controller is named as Dual CytoKontroller 1, and regulates M_1_ clearance μ_3_ and pathogen removal rate δ_3_ based on IL-6 cytokine level, according to [μ3,δ3]cont=[μ3,δ3](1+αIL−6nKdn+IL−6n)* . The second dual controller is named as Dual CytoKontroller 2, and regulates M_1_ clearance μ_3_ and M_1_ differentiation (i.e., M_2_ production rate) γ_3_ based on IL-6 cytokine level, according to [μ3,γ3]cont=[μ3,γ3](1+αIL−6nKdn+IL−6n)* . The third dual controller is named as Dual CytoKontroller 3, and regulates M_2_ production rate γ_3_ and pathogen removal rate δ_3_ based on IL-6 cytokine level, according to [γ3,δ3]cont=[γ3,δ3](1+αIL−6nKdn+IL−6n)* . The triple controller is named as Triple CytoKontroller, and regulates M_1_ clearance μ_3_, M_1_ differentiation γ_3_, and pathogen removal rate δ_3_ altogether, according to [μ3,δ3,γ3]cont=[μ3,δ3,γ3](1+αIL−6nKdn+IL−6n)* . Note that all these multiple species-based controllers have used the same model parameters as in CytoKontroller 1 without any customization for an optimized performance, for both a demonstration purpose and a direct comparison to the single species-based controllers.

Results in Figure 6 revealed that as long as the controller includes M_1_ clearance μ_3_ regulation, such as Dual CytoKontroller 1 and 2 and Triple CytoKontroller, it would offer a satisfactory performance, with a success rate of 89, 91, and 95%, respectively, even higher than the best-performing single species-based controller, CytoKontroller 1. In contrast, while Dual CytoKontroller 3 outperformed controllers that only regulate γ_3_ or δ_3_ (i.e., CytoKontroller 2 and 4), leaving out μ_3_ regulation seems to have undermined the performance, as compared to the ones that feature μ_3_ regulation, yielding a success rate of 72%.

**Figure 6:**
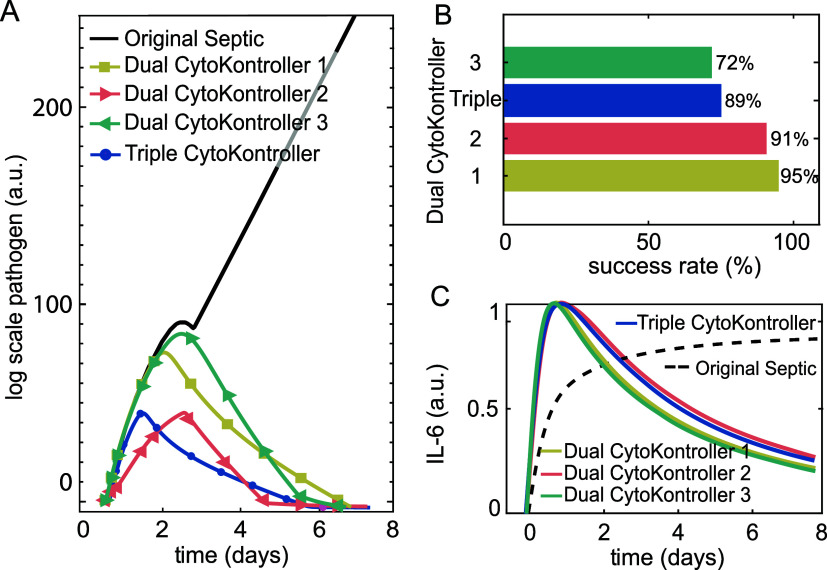
Dual and triple CytoKontroller performance evaluation. (A) Comparison of single log-scale pathogen dynamics from simulations with and without control. (B) Success rate in reverting the septic condition to acute healing with each CytoKontroller. (C) Comparison of single IL-6 dynamics from simulations with and without control. Black plots are for original septic simulation obtained from the global sensitivity analysis, and mustard, orange, green, and blue are for Dual CytoKontroller 1, 2, 3, and Triple CytoKontroller, respectively.

Altogether, these results confirmed the hypothesis that regulating multiple species could improve the controller performance, which makes sense from the control theory perspective, where increasing the number of manipulated variables would enhance the capability of controlling the system. Furthermore, regulating μ_3_ seems to be critical in ensuring the efficacy of the control, which is probably due to the important role of M_1_ in bridging pro- and anti-inflammatory response in the system, as well as its function in suppressing pathogen.

## Conclusion

Current sepsis treatment depends mainly on measures including source control, resuscitation, and palliative interventions.^43,44^ However, there exists a growing need for targeted therapies that focus on innovative approaches to address macrophage paralysis observed in sepsis. An effective septic therapeutic should regulate both the hyper- and hypo-inflammatory phases within a dysfunctional immune process, thereby preventing septicemia and averting additional organ malfunction.^22^ Here we demonstrated that systems biology modeling is invaluable for unraveling essential signaling pathways and devising potential controller motifs that can function as immune-modulating interventions, and feedback controllers hold great promise as effective interventions for sepsis.

Among the four single-species-based controllers, we observed that modulating M_1_ clearance or pathogen degradation had the most substantial efficacy, rescuing approximately 70 and 85% of the previously septic simulations. This suggests that targeting either the source of inflammation (pathogen) or the amplifier of inflammation (M_1_ macrophages) can offer robust points of intervention. In contrast, the M_2_-promoting controller (designed to accelerate resolution) had a minimal impact, suggesting that suppressing inflammation is more effective than promoting tissue repair after an immune dysregulation is established. Results with the multiple species-based controllers further suggest the complexity of the sepsis process that regulating multiple activities simultaneously could lead to further improvement of the regulation. This further suggests future work on controller designs that monitor multiple biomarkers for more accurate detection of the onset of sepsis and regulate multiple activities for enhanced efficacy.

Beyond its immediate applications, our deterministic reductionist ODE model provides a framework for developing complex immune models, designing controller motifs, and uncovering novel immune regulatory mechanisms. Rather than modeling the entire immune system, we demonstrated that targeting well-characterized pathways such as IL-6 and TGF-β signaling can inform effective therapeutic controller designs for sepsis. These findings will guide future biological regulators for sepsis and related immune disorders, supporting their experimental implementation and computational modeling of immune dynamics.

We also note that, while our ODE model captures the key immune dynamics, we noticed discrepancies between the fitted parameter values (e.g., γ_2_, γ_3_) from previously reported values such as in^23^. Detailed comparison is given in SI Table S1. While several reasons could have led to this observation, such as model complexity (e.g., simplification of details dynamics), model structure, and the experimental conditions, identifying precise kinetic parameter values is an involved process that requires customized experiments, in-depth model identification, etc., which we will investigate in future work.

## Supplementary Material



## Data Availability

The model script and codes used for model development and control analysis can be found on Zenodo: 10.5281/zenodo.17444427.

## Notes

Detailed model development and validation. Table of nominal model parameters. Figures on detailed results from local and global sensitivity analysis (PDF)
