# Supplementary material for: An In-Silico Study on the Design of Biological Controllers for Sepsis Regulation
Source: ACS Omega. 2026 Mar 11;11(11):17758–68. doi: 10.1021/acsomega.5c12020 (PMC13019193; doi:10.1021/acsomega.5c12020)
Supplement: Supplementary file 1 [file ao5c12020_si_001.pdf]

An *in-silico* Study on the Design of Biological Controllers for Sepsis Regulation

Supporting Information (SI)

Derrick Dankwa[†,1], Syeda Simra Shoaib[†,2], Leopold N. Green[*,1], Xun Tang[*,2]

[1] *Weldon School of Biomedical Engineering, Purdue University, West Lafayette, IN 47905*

[2] *Cain Department of Chemical Engineering, Louisiana State University, Baton Rouge, LA 70803*

[*] *Corresponding authors: xuntang@lsu.edu; greenln@purdue.edu*

1. Sepsis Model development

The mechanistic ODE model was developed around the key species in the system, following the law of mass action to describe macrophage polarization in sepsis. The 19 kinetic parameters were identified by fitting the model to reported cell count dynamics (neutrophil, M1, and M2) in Torres et al.[1] The nominal value for each parameter is provided in Table S1. Note that model parameter C_{max}, was obtained from Torres et al.[1] All simulations reported in this manuscript were performed with MATLAB R2022a, using the ode15s solver to solve the ODEs. For the global sensitivity analysis, we perturbed all the kinetic parameters range from 0.1 to 10 times their Nominal Values, except the M1 threshold θ_{eff}, to ensure its biological relevance.

In the Torres et al[1] experiments, a single intraperitoneal injection of 3% thioglycollate broth was administered to induce localized inflammation. Peritoneal exudate cells were then harvested every ten time points over a seven-day period, encompassing both the acute inflammatory phase and subsequent resolution. Neutrophils, inflammatory (M1-like) macrophages, and anti-inflammatory (M2-like) macrophages were quantified via flow cytometry using established surface marker profiles (Ly6G, CD11b, F4/80, Ly6C). The observation captured a characteristic self-resolving inflammatory response, marked by early neutrophil influx, delayed monocyte and macrophage recruitment, transient M1 macrophage dominance, and a subsequent shift toward M2 macrophages during resolution. This temporal profile aligns closely with the dynamics simulated in our model, making it a suitable benchmark for parameterization and qualitative validation. To obtain the parameter values, the model was fitted by minimizing the summed square errors between the predicted and the Torres et al. reported neutrophils, M1 and M2 macrophages cell counts over the course of eight days, using the trust region method within PottersWheel, a MATLAB toolbox for parameter estimation[2].

The initial guesses for most of the parameters were adopted or inferred from prior fitted parameters to related databases. Specifically, values for maximum number of cells (C_{max}), production rate of Neutrophil (α_1), M1-driven pathogen growth coefficient (α_2), differentiation rate from M1 to M2 (γ_3), Pathogen removal rate by Neutrophils (γ_4), recruitment rate of M0 (γ_5), M1 removal rate (μ_3), Pathogen degradation (δ_3) and Neutrophil removal rate (μ_1) were drawn from Torres et al[1]. Additional parameters including the M0 to M1 differentiation rate (γ_2), TGF-beta production rate (γ_1) and IL-6 degradation rate (δ_1) were sourced from Nagaraja et al.[3], who fitted *in vivo* and *in vitro* data from Newman et al.[4] , Fadok et al.[5], and Wong et al.[6] respectively; cytokine production rates were estimated as $k = \frac{C}{Mt}$, with linear regression applied where multiple measures were available, and degradation rates from half-life via $k = \frac{0.693}{t_{\frac{1}{2}}}$. Monocyte and M2 removal rates (μ_2 and μ_4) were obtained from Quintela et al.[7] with experimental values already fitted from Marchuk et al.[8] IL-6 production rate (β) and TGF-beta degradation rate (δ_2) from fitted values from Brady et al.[9] and Murphy et al.[10] respectively.

Table S1. Nominal Kinetic Parameters for Sepsis Model, related to STAR Method. Parameters were estimated by fitting the model to experimental data from a mouse model of peritonitis. Cell counts for neutrophils (N), M1, and M2 macrophages were measured in units of 10^7 cells from n mice. Simulated values for these cell types use the same unit, while others (pathogen, IL-6, and TGF-β) are in arbitrary units.

Description	Parameter	Value	Literature References	
hill function coefficient	n	5.41	-	-
hill function coefficient	K	0.75	-	-
threshold for pathogen regulation by M1	θ_{eff}	0.56 (10^7 cells)	-	-

maximum number of cells (not fitted)	C_{max}	10^7 cells*	10^7 cells	Torres et al.,[1]
production rate of Neutrophil	α_1	1.48 (10^7 cells day^{-1})	15.89 (N $units^{-1}$ day^{-1})	
M1-driven pathogen growth coefficient	α_2	13.05 ($a.u^{-1}$ day^{-1})	35	
production rate of TGF-beta	γ_1	21.89 ($a.u^{-1}$ day^{-1})	4.5×10^{-5} (ng $cell^{-1}$ day^{-1})	Nagaraja et al.,[3]
differentiation rate from M0 to M1	γ_2	69.55 (day^{-1})	2.4 ($M\text{-}units^{-1}$ day^{-1})	
differentiation rate from M1 to M2	γ_3	20 ($a.u^{-1}$ day^{-1})	8.281 ($M\text{-}units^{-1}$ day^{-1})	Torres et al.,[1]
Pathogen removal rate by Neutrophils	γ_4	5 ($a.u^{-1}$ day^{-1})	0.295 ($N\text{-}units^{-1}$ day^{-1})	
recruitment rate of M0	γ_5	9.51 (a.u 10^7 $cells^{-1}$ day^{-1})	0.24 (day^{-1})	
Neutrophil removal cell	μ_1	0.07 (day^{-1})	3.97 (day^{-1})	
Monocyte removal cell	μ_2	2.75 (day^{-1})	5.15 (day^{-1})	Quintela et al.[7]
M1 removal cell	μ_3	2 (day^{-1})	6.95 (day^{-1})	Torres et al.[1]
M2 removal cell	μ_4	0.74 (day^{-1})	8.27 (day^{-1})	Quintela et al.[7]
IL-6 production rate	β	0.42 (a.u 10^7 $cells^{-1}$ day^{-1})	0.6 (cytokine $cell^{-1}$$day^{-1}$)	Brady et al.[9]
IL-6 degradation rate	δ_1	0.82 (day^{-1})	11.08 (day^{-1})	Nagaraja et al.[3]
TGF-beta degradation rate	δ_2	25.80 (day^{-1})	0.35 (day^{-1})	Murphy et al.[44]
Pathogen degradation	δ_3	7.47(day^{-1})	0.5 (day^{-1})	Torres et al.[1]
Slope for effector function	κ	17.69(10^7 cells)	-	-
*Note that, for C_{max}, we have used 1000 in the simulation, but the output cell counts have been normalized to have a unit of per 10^7, same as the in Torres et al.[1]				

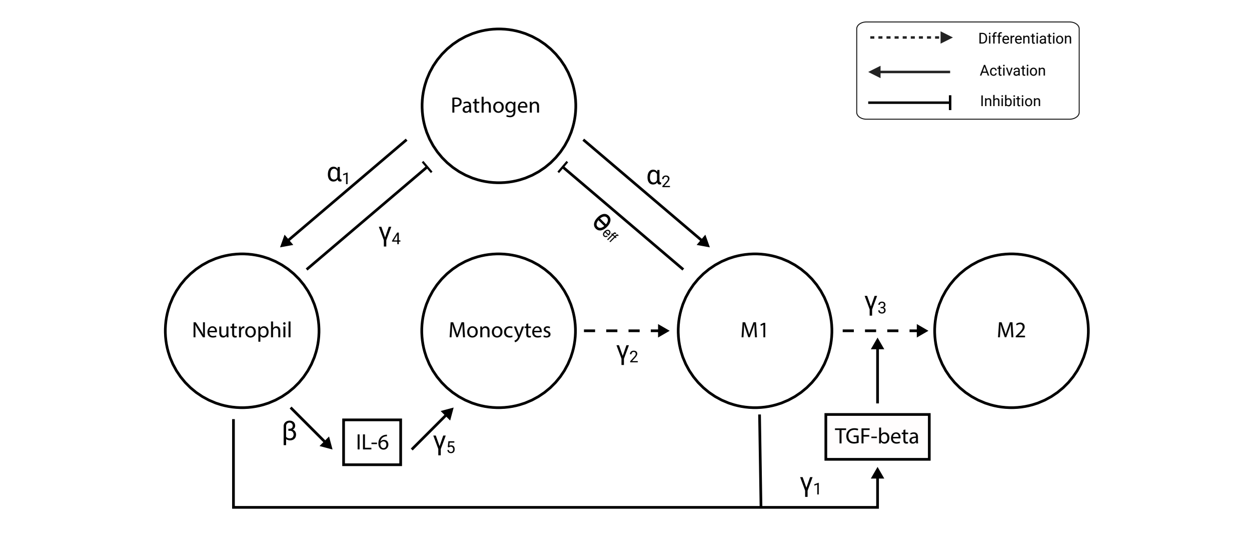

Figure S1. Diagram showing the interactions in the ODE-based model. Dashed arrow indicates cell differentiation, solid arrow indicates activation function, and arrow with and end vertical indicates inhibition function.

2. Controller Performance Evaluation

Figure S2-S9 summarize the performance of each of the eight CytoKontrollers, in terms of individual cell species and cytokines dynamics over time, illustrating the differences between the regulated and the unregulated septic dynamics.

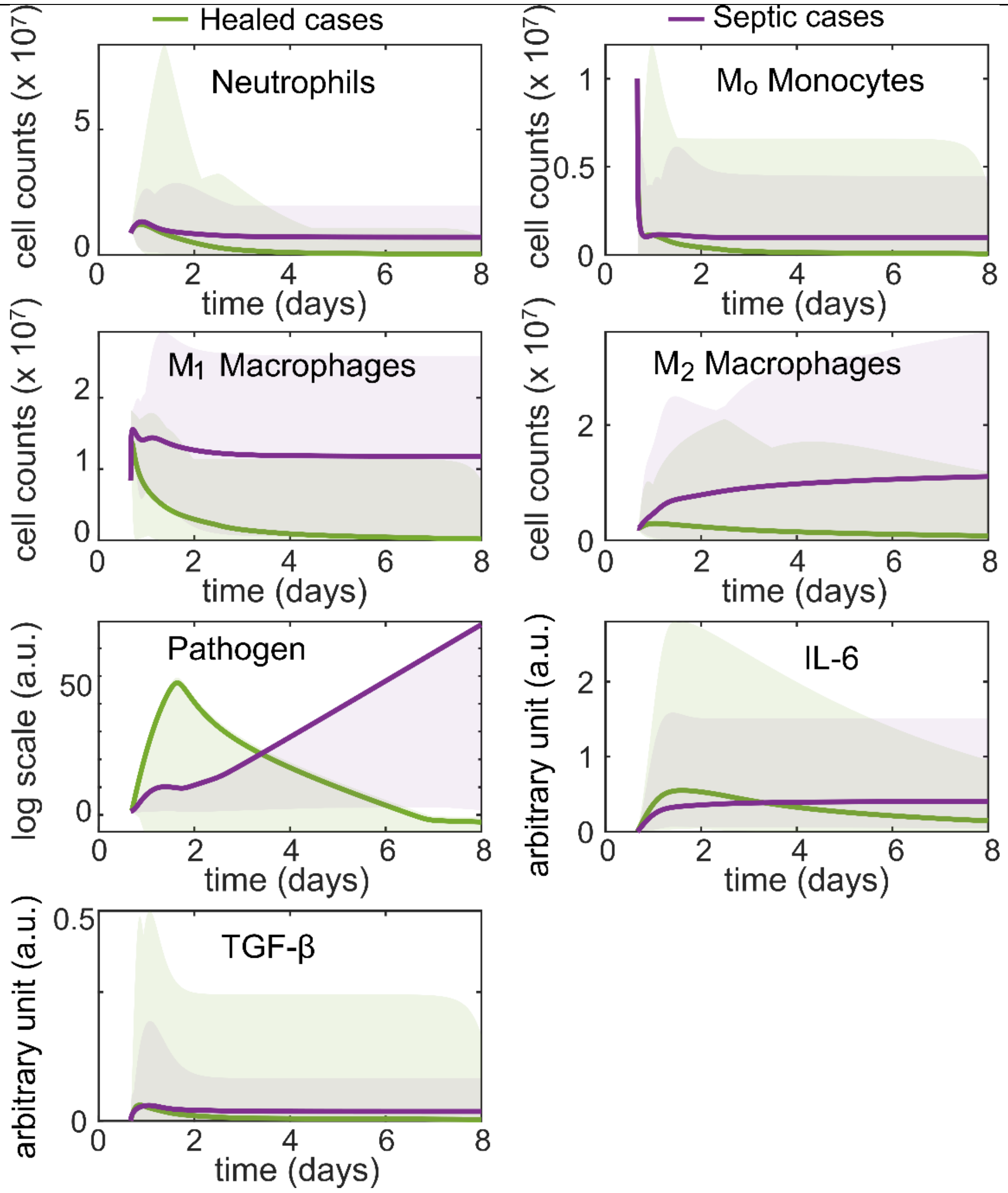

Figure S2. CytoKontroller 1 (M1) Performance on Pathogen Clearance.

Comparison of healed (light green) and septic cases (purple) conditions demonstrates significant improvement in pathogen clearance and immune regulation under control intervention. Thicker plots represent the average trajectories; thinner plots are individual simulations.

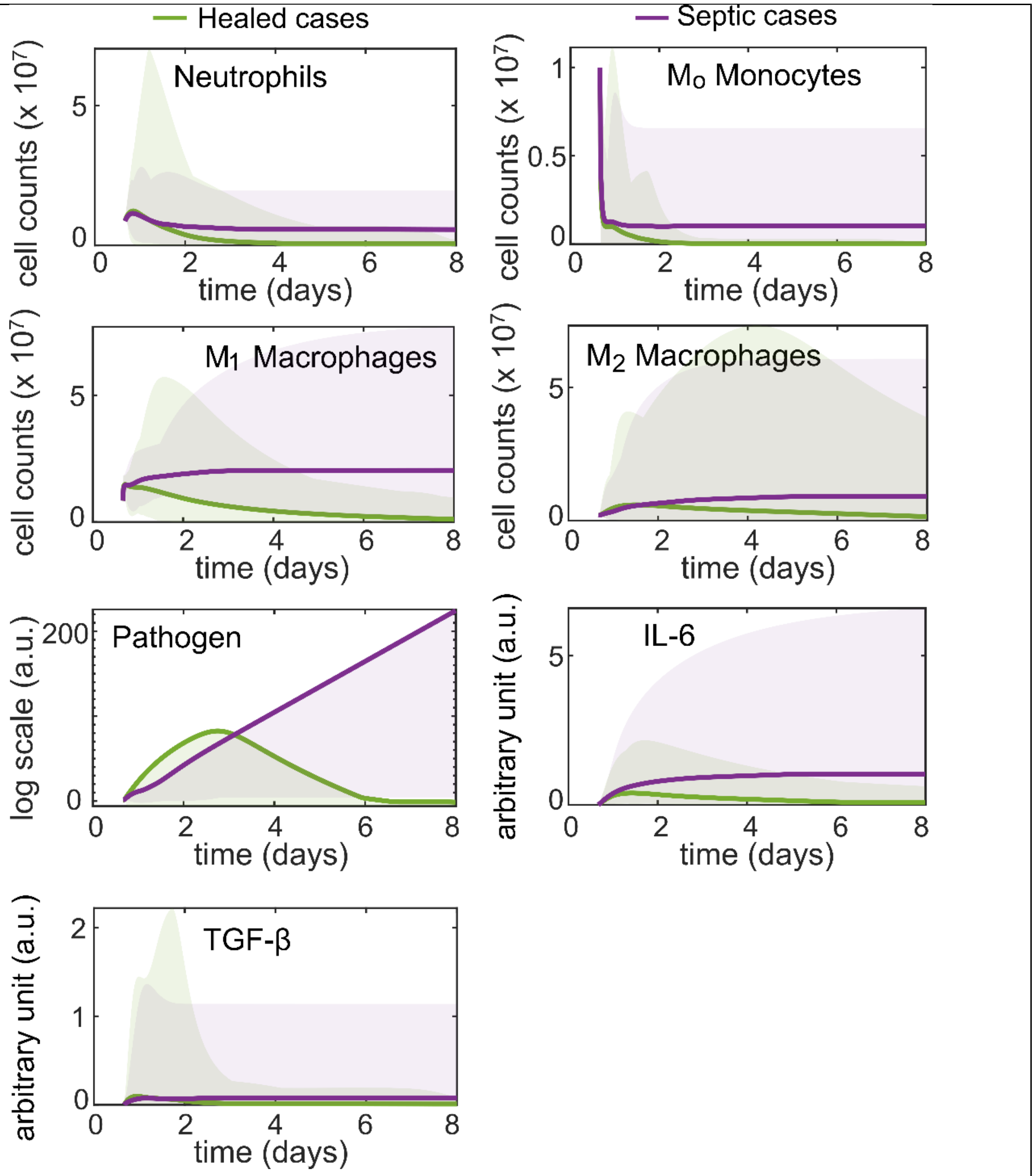

Figure S3. CytoKontroller 2 (Pathogen) Performance on Pathogen Clearance. Comparison of controlled (green) and septic (purple) conditions demonstrates significant improvement in pathogen clearance and immune regulation under control intervention.

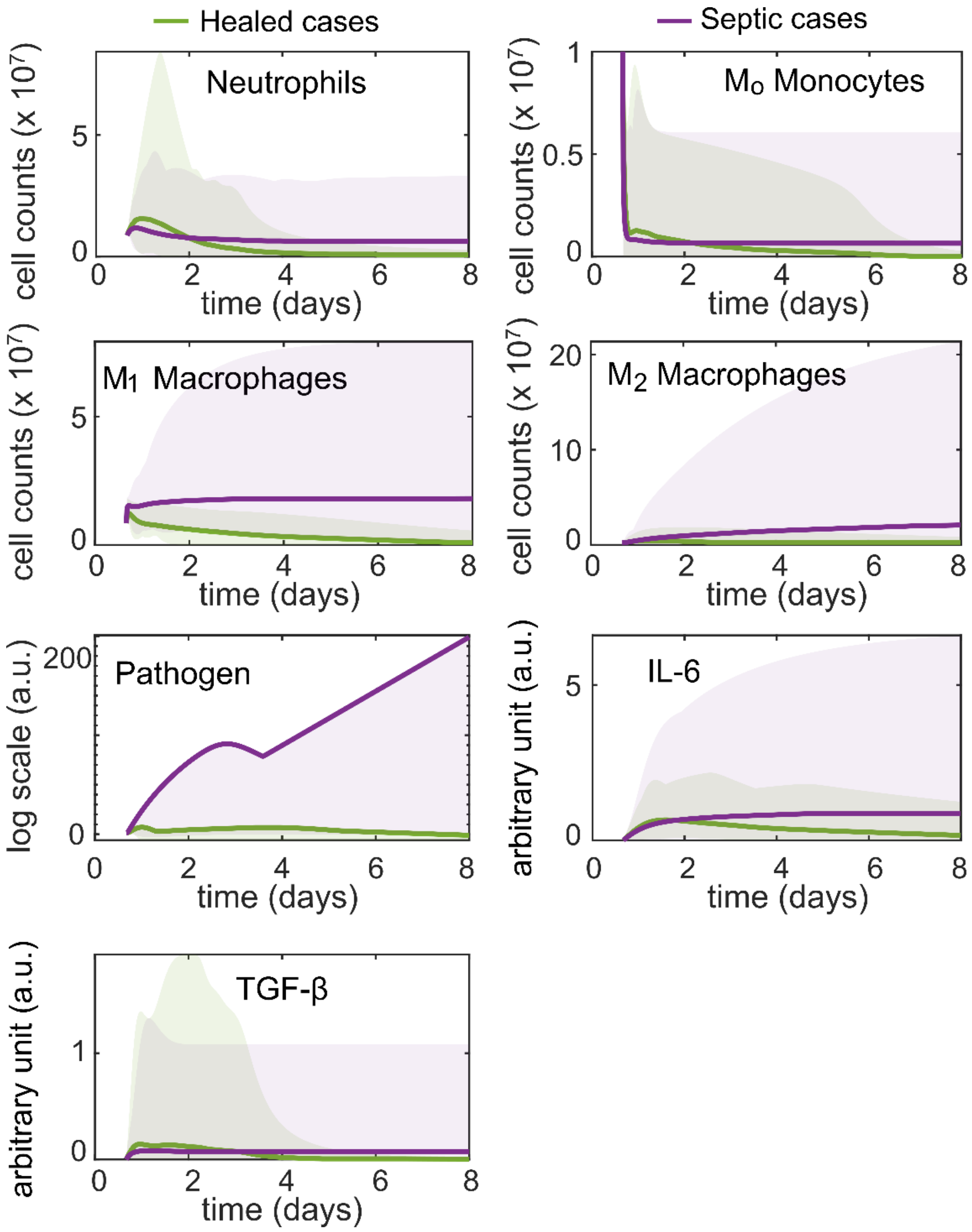

Figure S4. CytoKontroller 3 (M_o) Performance on Pathogen Clearance. Comparison of controlled (green) and septic (purple) conditions demonstrates significant improvement in pathogen clearance and immune regulation under control intervention.

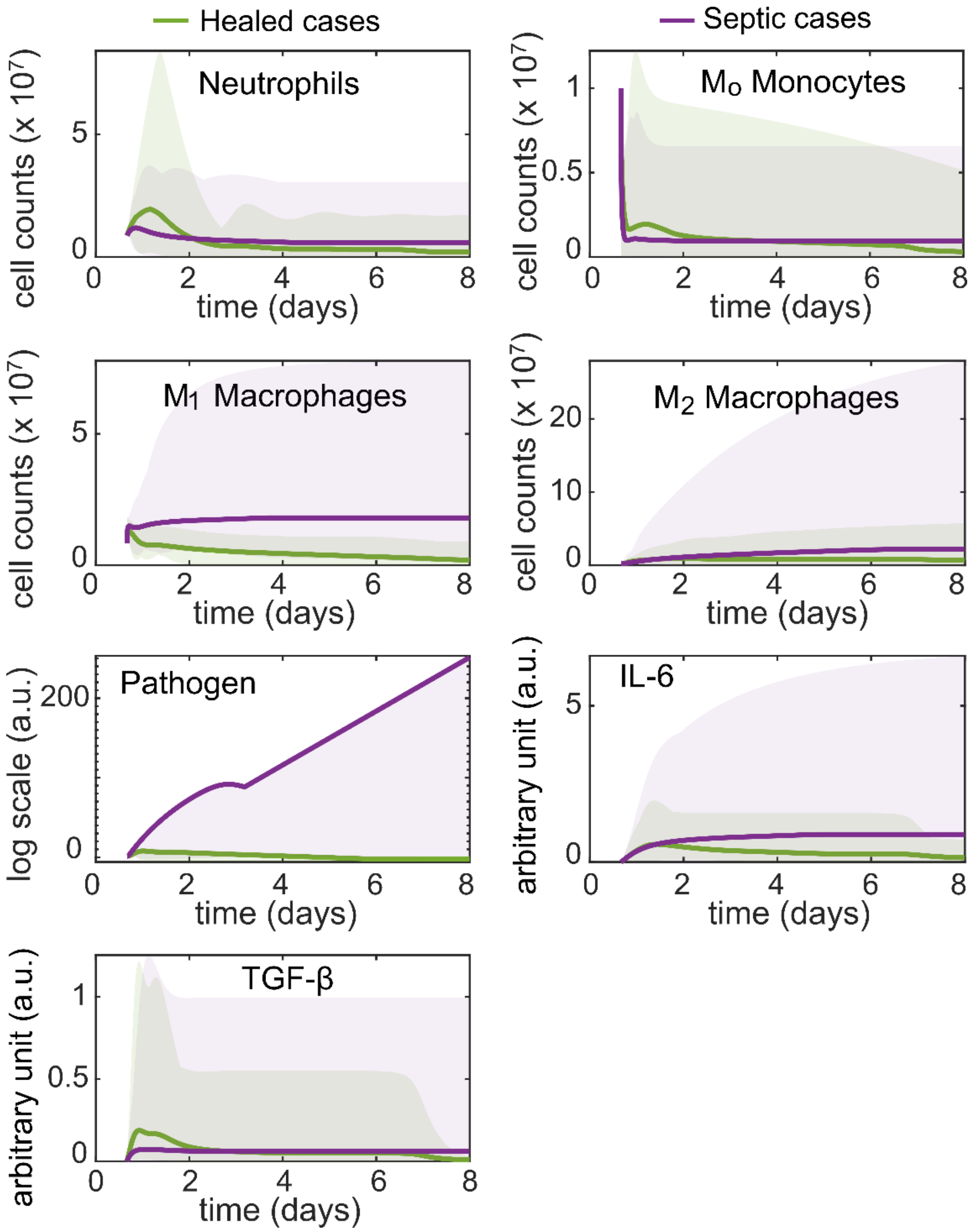

Figure S5. CytoKontroller 4 (M2) Performance on Pathogen Clearance. Comparison of controlled (green) and septic (purple) conditions demonstrates significant improvement in pathogen clearance and immune regulation under control intervention.

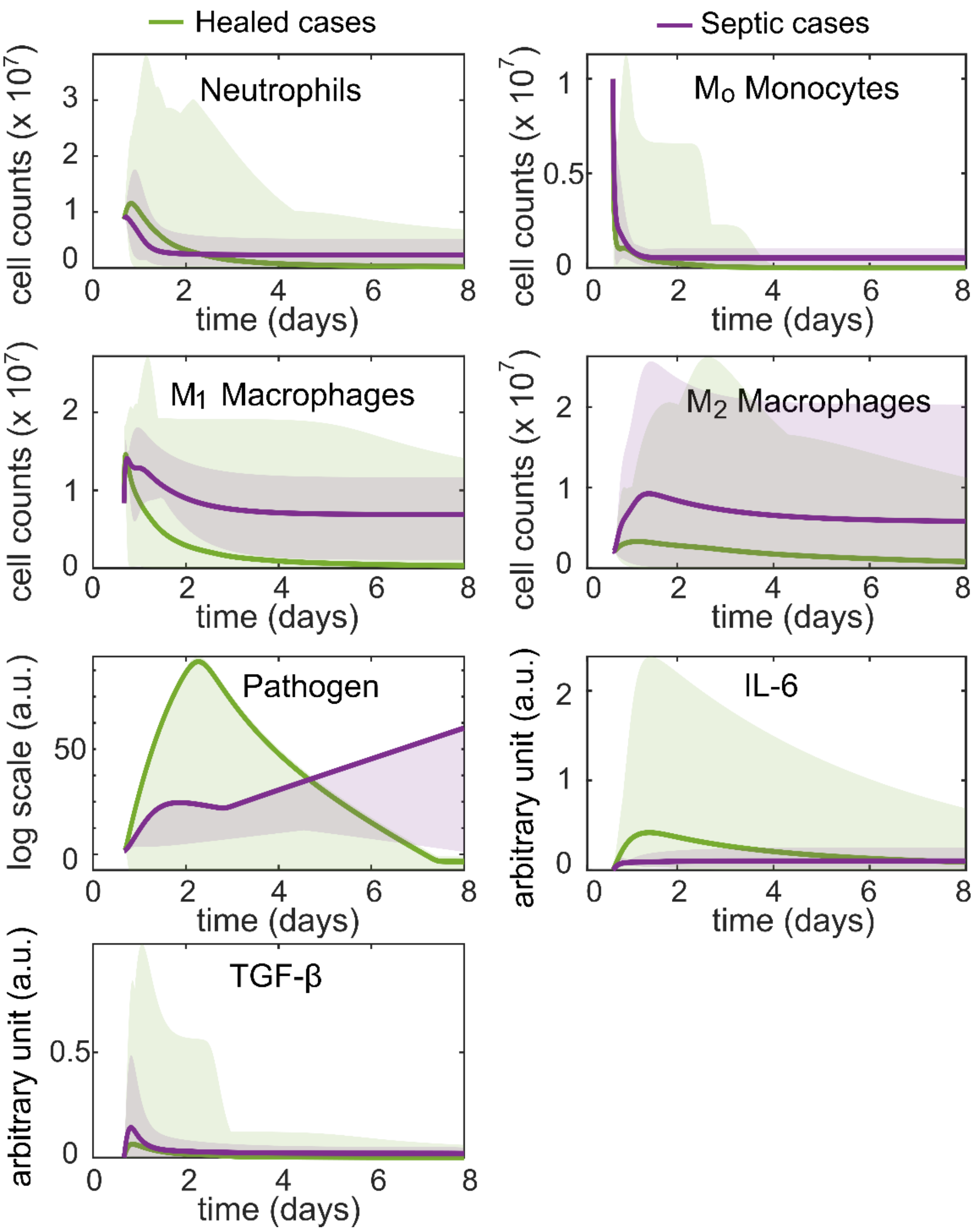

Figure S6. Dual CytoKontroller 1 (M1 and P) Performance on Pathogen Clearance. Comparison of controlled (green) and septic (purple) conditions demonstrates significant improvement in pathogen clearance and immune regulation under control intervention.

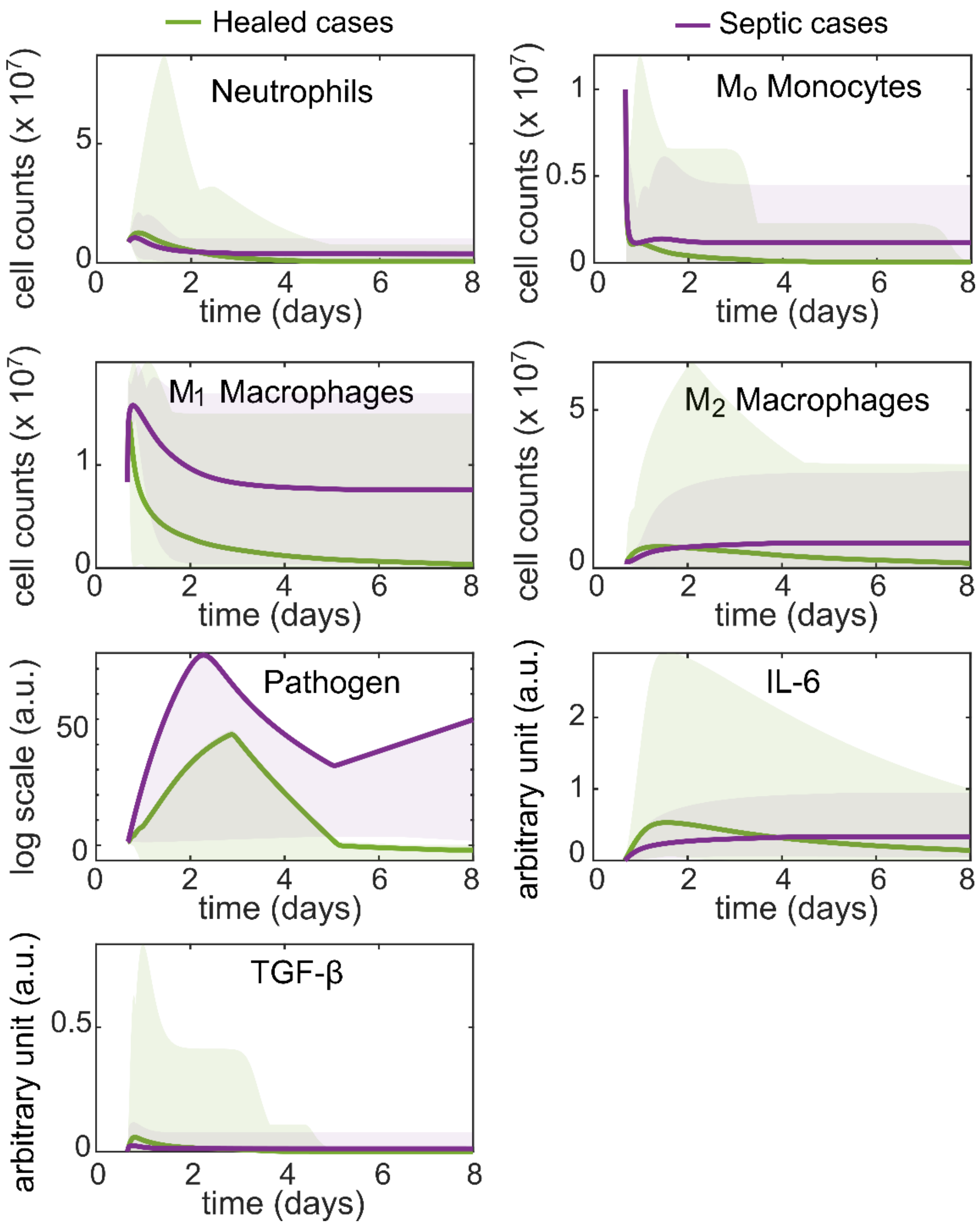

Figure S7. Dual CytoKontroller 2 (M1 and M2) Performance on Pathogen Clearance. Comparison of controlled (green) and septic (purple) conditions demonstrates significant improvement in pathogen clearance and immune regulation under control intervention.

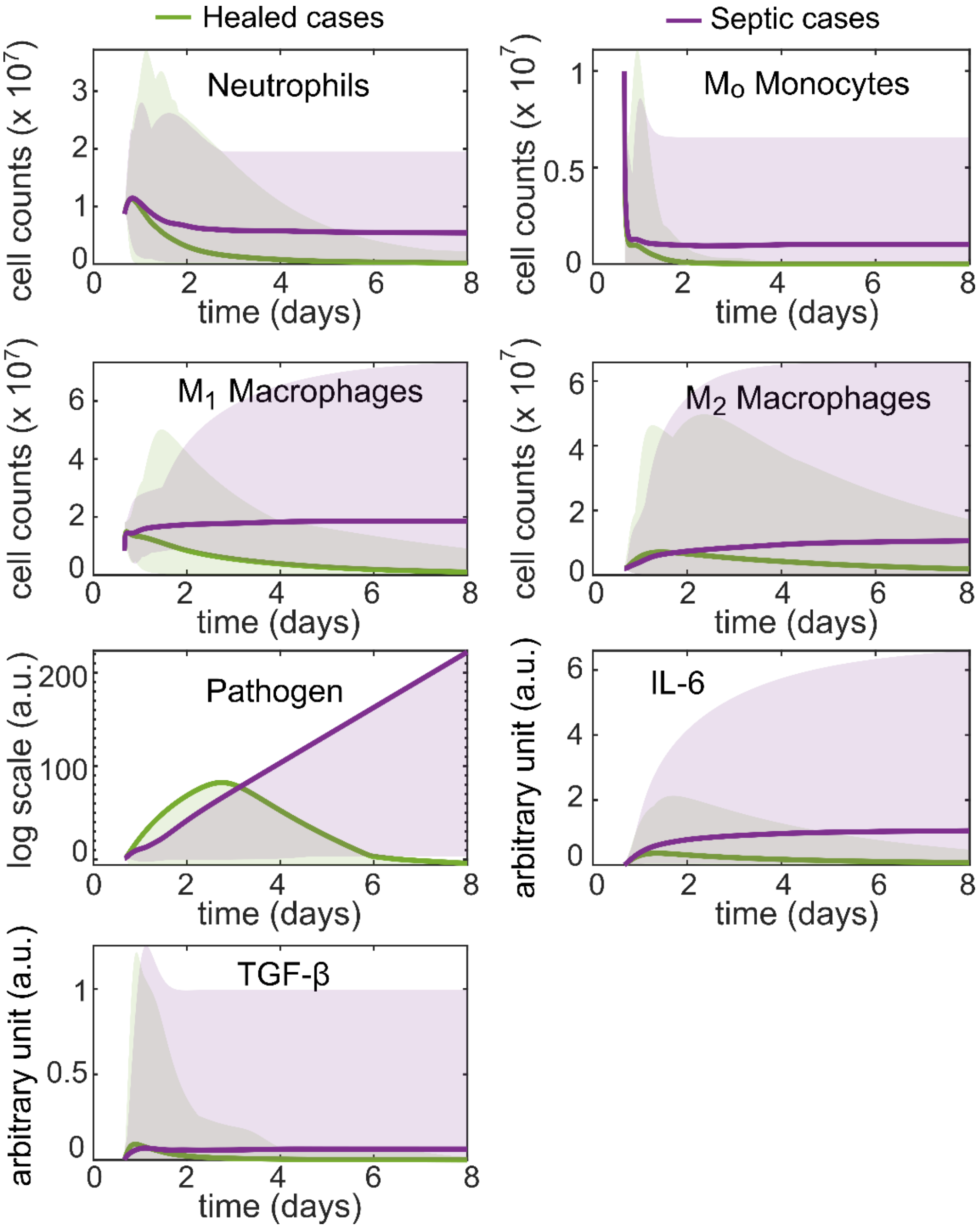

Figure S8. Dual CytoKontroller 3 (M2 and P) Performance on Pathogen Clearance. Comparison of controlled (green) and septic (purple) conditions demonstrates significant improvement in pathogen clearance and immune regulation under control intervention.

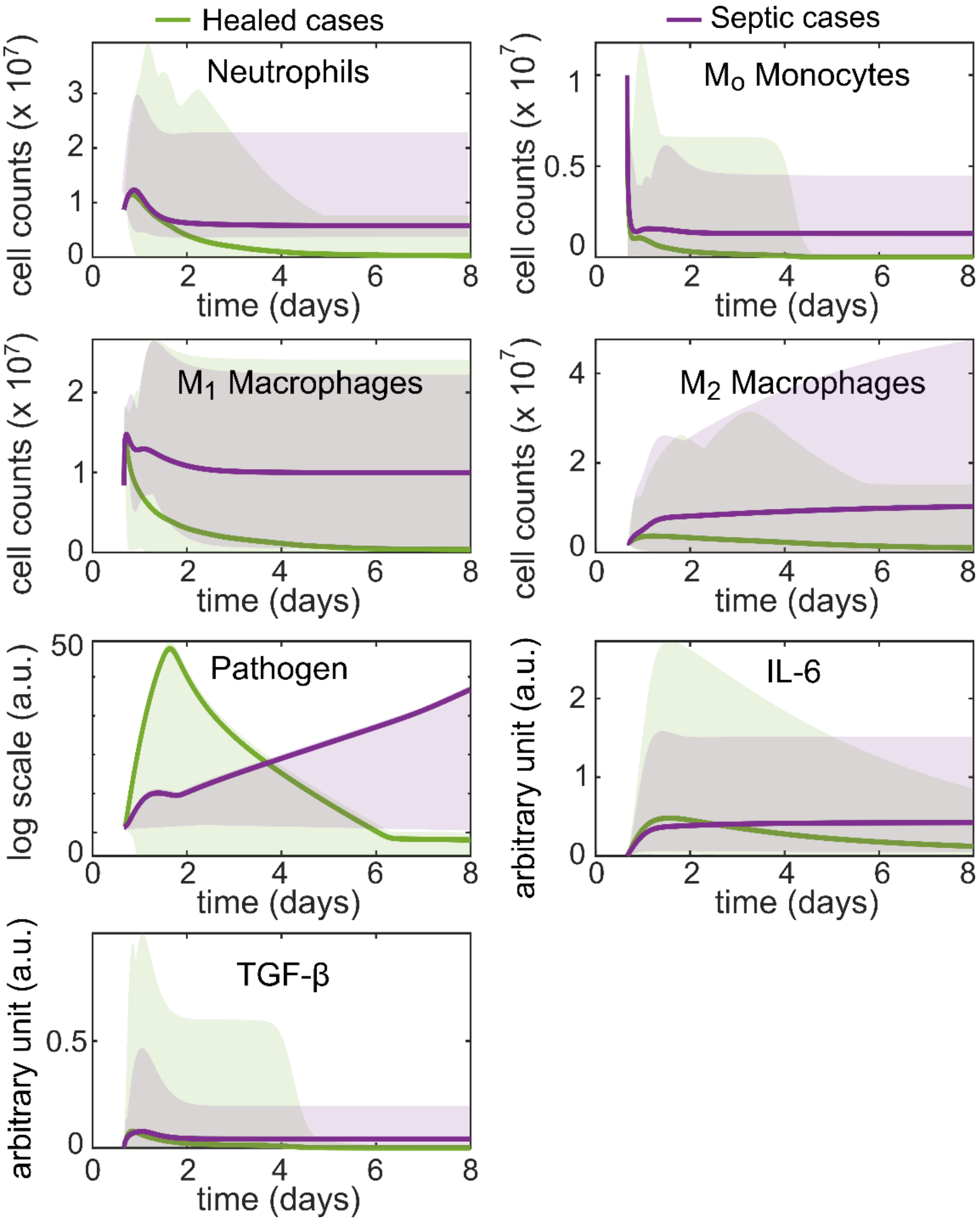

Figure S9. Triple CytoKontroller (M1, M2 and P) Performance on Pathogen Clearance. Comparison of controlled (green) and septic (purple) conditions demonstrates significant improvement in pathogen clearance and immune regulation under control intervention.

3. Local Sensitivity Analysis

The local sensitivity analysis evaluates the model's sensitivity to single parameter variation and provides insights into the most prominent parameters that can serve as the regulation targets. In our study, we independently perturbed each of the 18 kinetic parameters to [0.1, 0.5, 2, 10]*$Nominal\ Value$, and used the following three metrics to quantify the significance of the impact of each parameter: Endpoint Cell Counts, Maximum Cell Counts, and Time to Peak. Specifically, the "Endpoint Cell Count" quantifies the abundance of each cell species at the end of the 8-day simulation, the "Maximum Cell Count" of each species and the "Time to Peak" metrics complement the "Endpoint Cell Count" by gauging the temporal signal and cellular dynamics, to distinguish between acute and septic healing conditions.

In general, parameters $\mu_3, \mu_4, \delta_1, \gamma_3, \beta, \delta_3$ showed more impact on all the cell types as compared to other parameters, as these parameters affect either the generation or consumption of IL-6 or the cell concentration directly. Therefore, when we tested the controller robustness, we chose to perturb these parameters.

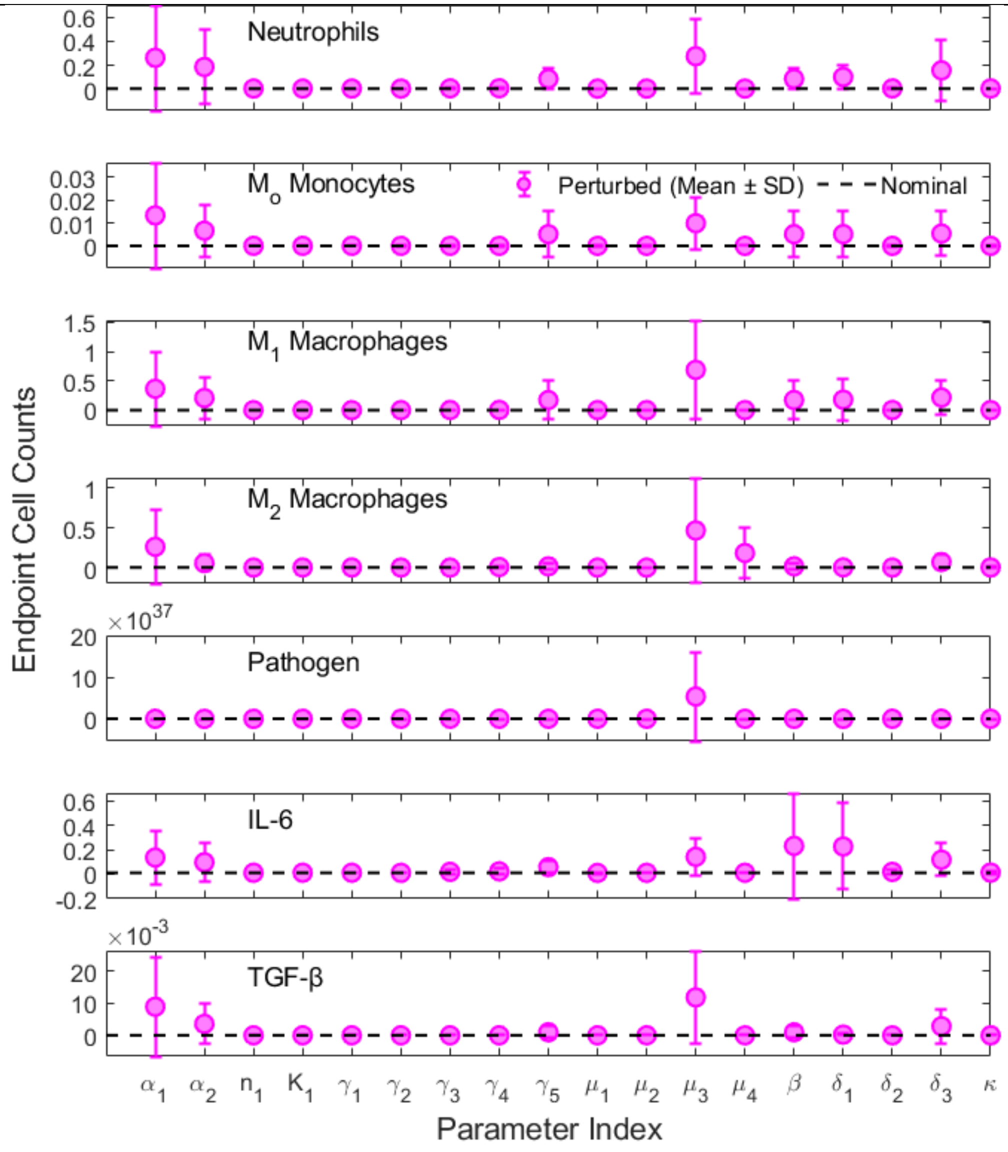

Figure S10. Local Sensitivity Analysis of Endpoint Concentrations Identifies Key Kinetic Regulators. This figure presents time to peak for immune cells, pathogen load, and inflammatory mediators, as determined by a local sensitivity analysis.The circles represent the average endpoint values across these adjustments, while the error bars show the standard deviation. The dashed black line indicates the trajectory without any perturbations. This analysis reveals which parameters have the most significant impact on infection outcomes, highlighting the main drivers of the interactions between immune responses and pathogens.

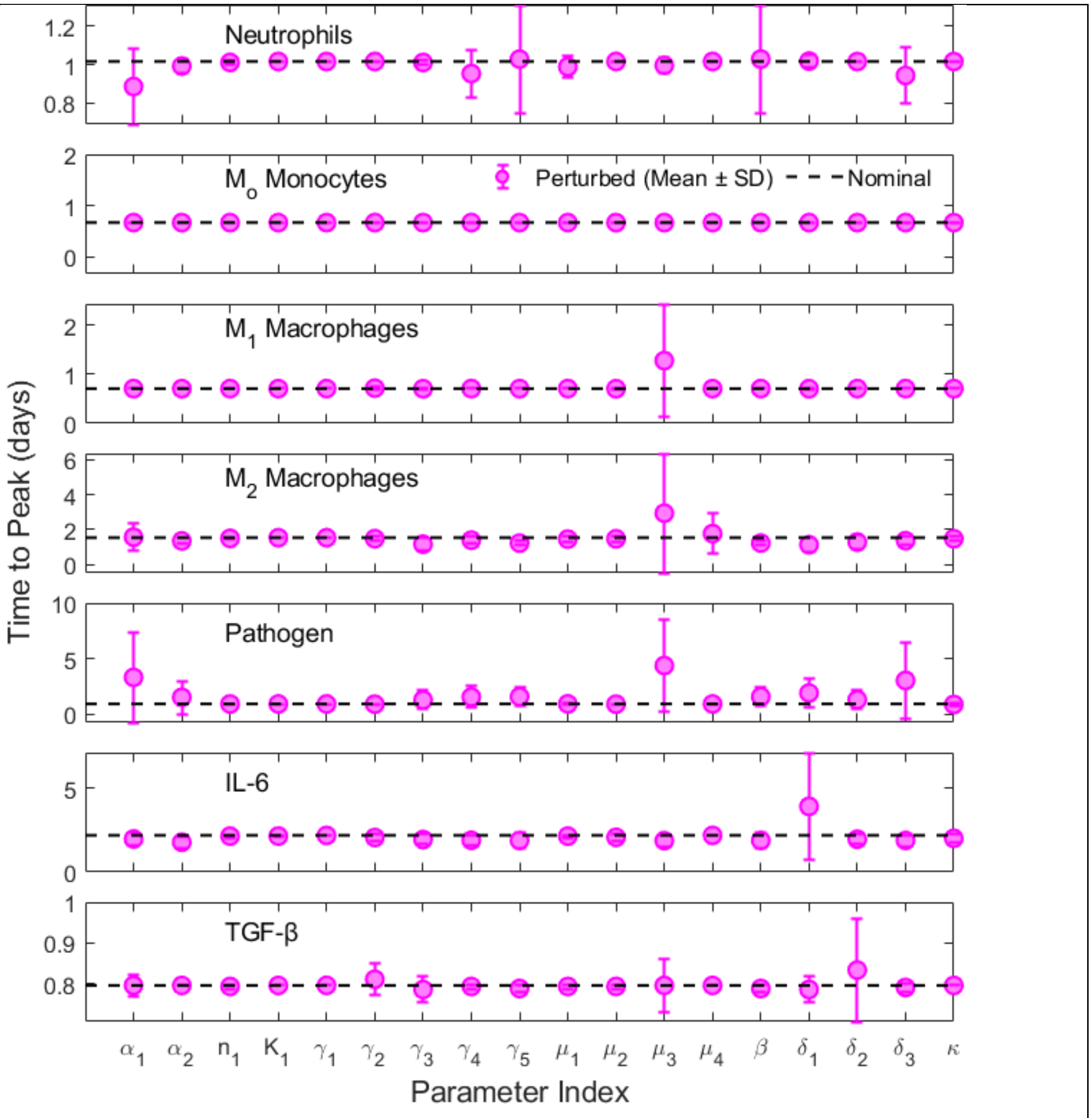

Figure S11. Local Sensitivity Analysis of Time to Peak. This figure presents the values of immune cells, pathogen load, and inflammatory mediators measured on day 8, as determined by a local sensitivity analysis.The circles represent the average endpoint values across these adjustments, while the error bars show the standard deviation. The dashed black line indicates the trajectory without any perturbations. This analysis reveals which parameters have the most significant impact on infection outcomes, highlighting the main drivers of the interactions between immune responses and pathogens.

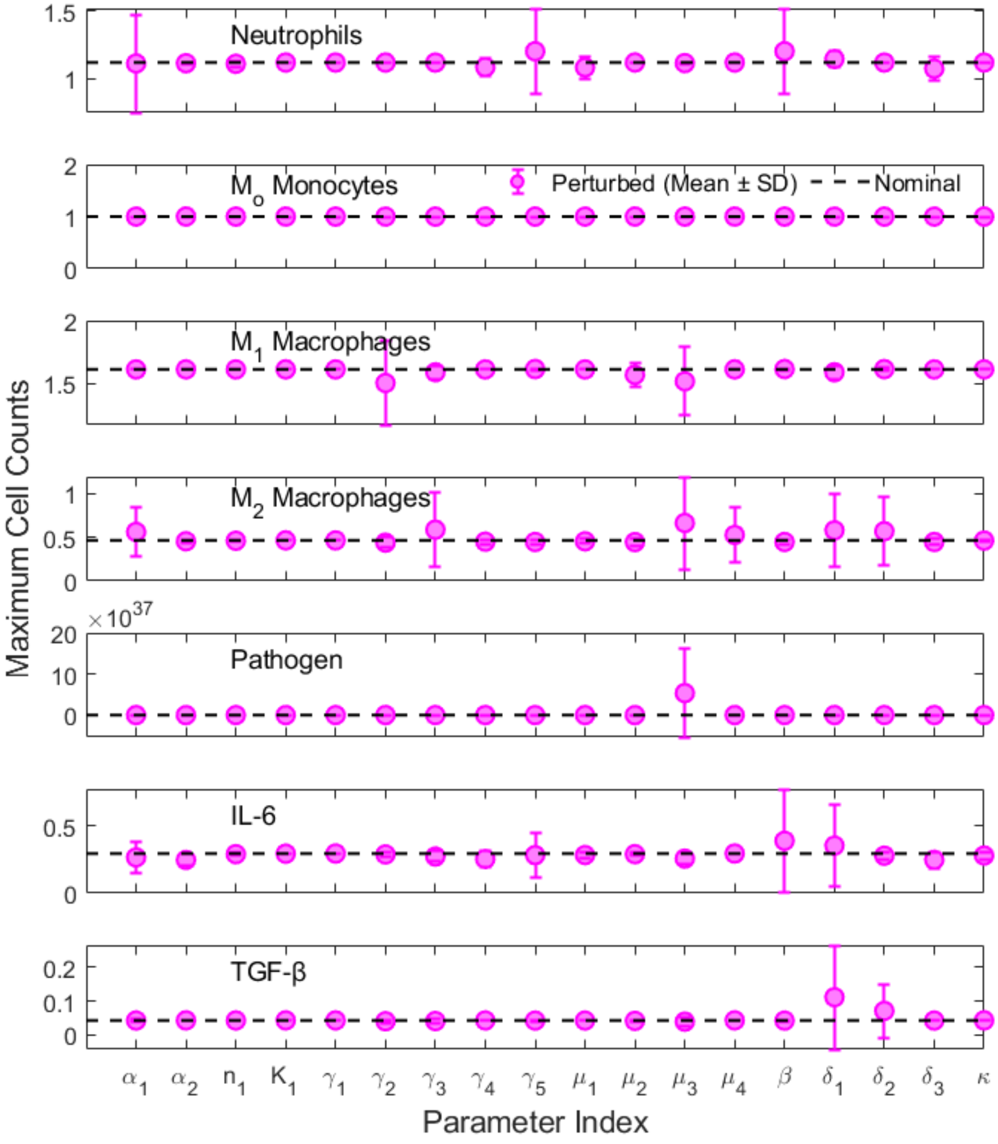

Figure S12. Local Sensitivity Analysis at Maximum Cell Counts. This figure presents maximum concetration for immune cells, pathogen load, and inflammatory mediators achieved in total 8 day simulation, as determined by a local sensitivity analysis.The circles represent the average endpoint values across these adjustments, while the error bars show the standard deviation. The dashed black line indicates the trajectory without any perturbations. This analysis

reveals which parameters have the most significant impact on infection outcomes, highlighting the main drivers of the interactions between immune responses and pathogens.

4. Global Sensitivity Analysis

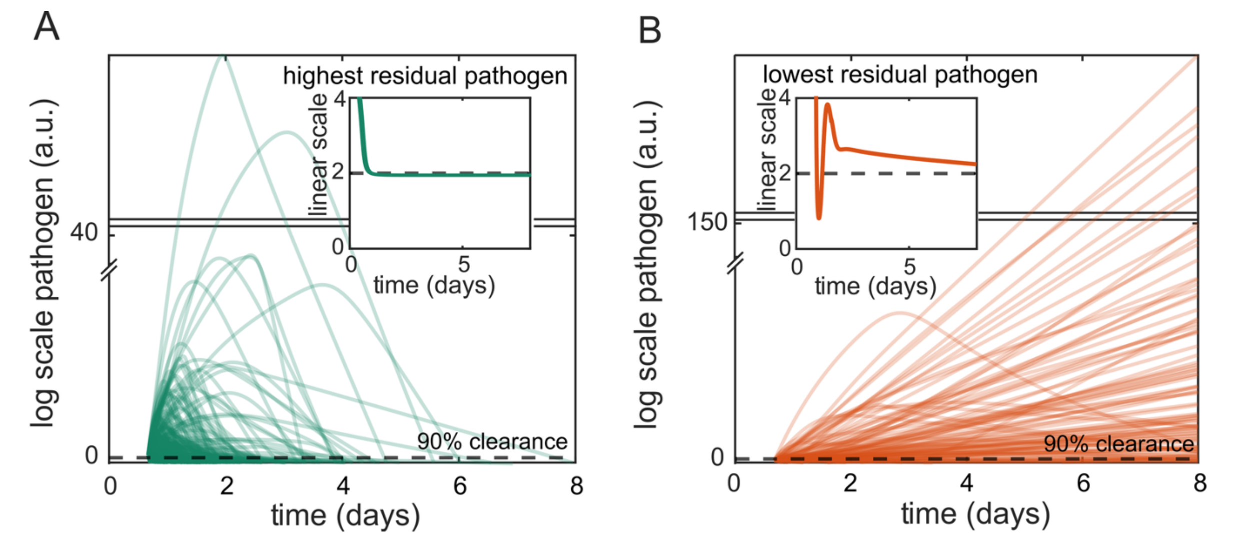

Figure S13. Global sensitivity analysis reveals distinct pathogen dynamics in acute (green) and septic (red) healing. (A) All acute trajectories are shown as light green lines, and the inset shows the trajectory with the highest pathogen level at the end of the simulation. (B) All septic trajectories are shown as light red lines, and the inset shows the trajectory with the lowest pathogen level at the end of the simulation. Main figure y-axes are in log-scale to capture the full dynamic range, and the insets y-axes are in linear scale for better illustration.

We note that some simulations in our study produced exceptionally high pathogen levels (e.g., Figure S13) that are unlikely to reflect biological reality. Several factors may have contributed to this outcome. First, the pathogen in the simulations is expressed in arbitrary units and does not correspond to actual cell or bacterial counts. Second, the simulations were generated using randomly perturbed model parameters that may yield biologically infeasible parameter combinations, resulting in unrealistically elevated pathogen levels. As the primary objective of

this simulation-based analysis is to elucidate differences between septic and acute conditions, the biological feasibility of all parameter combinations was treated as a secondary consideration.

References

(1) Torres, M.; Wang, J.; Yannie, P. J.; Ghosh, S.; Segal, R. A.; Reynolds, A. M. Identifying Important Parameters in the Inflammatory Process with a Mathematical Model of Immune Cell Influx and Macrophage Polarization. *PLOS Comput. Biol.* **2019**, *15* (7), e1007172. https://doi.org/10.1371/journal.pcbi.1007172.

(2) Maiwald, T.; Timmer, J. Dynamical Modeling and Multi-Experiment Fitting with PottersWheel. *Bioinformatics* **2008**, *24* (18), 2037–2043. https://doi.org/10.1093/bioinformatics/btn350.

(3) Nagaraja, S.; Wallqvist, A.; Reifman, J.; Mitrophanov, A. Y. Computational Approach To Characterize Causative Factors and Molecular Indicators of Chronic Wound Inflammation. *J. Immunol.* **2014**, *192* (4), 1824–1834. https://doi.org/10.4049/jimmunol.1302481.

(4) Newman, S. L.; Henson, J. E.; Henson, P. M. Phagocytosis of Senescent Neutrophils by Human Monocyte-Derived Macrophages and Rabbit Inflammatory Macrophages. *J. Exp. Med.* **1982**, *156* (2), 430–442. https://doi.org/10.1084/jem.156.2.430.

(5) Fadok, V. A.; Bratton, D. L.; Konowal, A.; Freed, P. W.; Westcott, J. Y.; Henson, P. M. Macrophages That Have Ingested Apoptotic Cells in Vitro Inhibit Proinflammatory Cytokine Production through Autocrine/Paracrine Mechanisms Involving TGF-Beta, PGE2, and PAF. *J. Clin. Invest.* **1998**, *101* (4), 890–898. https://doi.org/10.1172/JCI1112.

(6) Wong, S.; Schwartz, R. C.; Pestka, J. J. Superinduction of TNF-Alpha and IL-6 in Macrophages by Vomitoxin (Deoxynivalenol) Modulated by mRNA Stabilization. *Toxicology* **2001**, *161* (1–2), 139–149. https://doi.org/10.1016/s0300-483x(01)00331-6.

(7) Quintela, B. de M.; dos Santos, R. W.; Lobosco, M. On the Coupling of Two Models of the Human Immune Response to an Antigen. *BioMed Res. Int.* **2014**, *2014*, 410457. https://doi.org/10.1155/2014/410457.

(8) Marchuk, G. I. Identification of Parameters of Models. In *Mathematical Modelling of Immune Response in Infectious Diseases*; Marchuk, G. I., Ed.; Springer Netherlands: Dordrecht, 1997; pp 150–198. https://doi.org/10.1007/978-94-015-8798-3_6.

(9) Brady, R.; Frank-Ito, D. O.; Tran, H. T.; Janum, S.; Møller, K.; Brix, S.; Ottesen, J. T.; Mehlsen, J.; Olufsen, M. S. Personalized Mathematical Model Predicting Endotoxin-Induced Inflammatory Responses in Young Men. arXiv September 6, 2016. https://doi.org/10.48550/arXiv.1609.01570.

(10) Murphy, K. E.; McCue, S. W.; McElwain, D. L. S. Clinical Strategies for the Alleviation of Contractures from a Predictive Mathematical Model of Dermal Repair. *Wound Repair Regen.* **2012**, *20* (2), 194–202. https://doi.org/10.1111/j.1524-475X.2012.00775.x.
